# Use of the reversible jump Markov chain Monte Carlo algorithm to select multiplicative terms in the AMMI-Bayesian model

**DOI:** 10.1371/journal.pone.0279537

**Published:** 2023-01-03

**Authors:** Carlos Pereira da Silva, Cristian Tiago Erazo Mendes, Alessandra Querino da Silva, Luciano Antonio de Oliveira, Renzo Garcia Von Pinho, Marcio Balestre

**Affiliations:** 1 Department of Statistics (DES), Institute of Exact Sciences, Federal University of Lavras, Lavras, Minas Gerais, Brazil; 2 Faculty of Exact Sciences and Technology (FACET), Federal University of Grande Dourados, Dourados, Mato Grosso do Sul, Brazil; 3 Department of Agriculture (DAG), School of Agricultural Sciences, Federal University of Lavras, Lavras, Minas Gerais, Brazil; University Politehnica of Bucharest, ROMANIA

## Abstract

The model selection stage has become a central theme in applying the additive main effects and multiplicative interaction (AMMI) model to determine the optimal number of bilinear components to be retained to describe the genotype-by-environment interaction (GEI). In the Bayesian context, this problem has been addressed by using information criteria and the Bayes factor. However, these procedures are computationally intensive, making their application unfeasible when the model’s parametric space is large. A Bayesian analysis of the AMMI model was conducted using the Reversible Jump algorithm (RJMCMC) to determine the number of multiplicative terms needed to explain the GEI pattern. Three a priori distributions were assigned for the singular value scale parameter under different justifications, namely: i) the insufficient reason principle (uniform); ii) the invariance principle (Jeffreys’ prior) and iii) the maximum entropy principle. Simulated and real data were used to exemplify the method. An evaluation of the predictive ability of models for simulated data was conducted and indicated that the AMMI analysis, in general, was robust, and models adjusted by the Reversible Jump method were superior to those in which sampling was performed only by the Gibbs sampler. In addition, the RJMCMC showed greater feasibility since the selection and estimation of parameters are carried out concurrently in the same sampling algorithm, being more attractive in terms of computational time. The use of the maximum entropy principle makes the analysis more flexible, avoiding the use of procedures for correcting prior degrees of freedom and obtaining improper posterior marginal distributions.

## Introduction

In plant breeding programs, the selection and recommendation of superior genotypes are of considerable importance. The observed performance (phenotype) is determined by the genetic composition of the plant (genotype), by the conditions under which it develops (environment), and the interaction between these two factors, i.e. genotype×environment interaction (GEI) [1, 2]. GEI reduces the predictability of the performance of varieties in environments, making it difficult for the breeder to make a broad recommendation of genotypes. Moreover, efficient modeling of GEI has become a central aspect in the statistical analysis of data from multi-environmental trials (MET) [3]. Therefore, interest in the development of statistical methods aimed at identifying stable genotypes and satisfactory combinations between genotypes and environments is increasing. These methods aim to explore the positive effect of GEI for specific recommendations.

Linear-bilinear models are useful for evaluating genotype responses in different environments. Specifically, the additive main effects and multiplicative interaction (AMMI) model deserves special mention due to its wide applicability in the study of GEI by plant researchers and breeders [4, 5]. However, limitations in the standard analysis of the AMMI model (which considers parameters with fixed effects), such as difficulty in dealing with unbalanced data and heterogeneity of variance, have motivated the pursuit of more flexible methods.

An option to overcome such limitations is the use of the Bayesian paradigm, which allows the incorporation of more complex structures in the GEI analysis [6–8]. The first Bayesian proposals of the AMMI model were presented by Viele and Srinivasan [9] and Liu [10]. These authors presented an MCMC (Markov chain Monte Carlo) algorithm to correctly sample the model parameters, especially for those that describe GEI, whose support for the posterior joint distribution is not trivial. The flexibility of the Bayesian-AMMI in incorporating inference to the multiplicative terms of the biplot, random effects for genotypes, and historical information to experiments was illustrated by Crossa et al. [11], Perez-Elizalde et al. [12], and Oliveira et al. [13]. In turn, Romão et al. [14] and Silva et al. [15], empirically demonstrated the robustness of the Bayesian-AMMI model under unbalance and heterogeneity of variances between environments.

An aspect that is central in research involving AMMI analysis is the adjustment of the model with respect to the number of bilinear terms to be retained for explaining GEI. This issue has been widely explored in the frequentist context, and involves the use of both *F*-approximate tests and non-parametric procedures based on intensive computation [16–23]. In contrast, the selection of multiplicative terms in Bayesian-AMMI was done based on information criteria, such as Akaike’s Information Criterion (AIC), Bayesian Information Criterion (BIC), and Bayes Factor (BF) [10, 12].

Silva et al. [24] proposed a Bayesian version of the frequentist Shrinkage method published by Cornelius and Crossa [25], assigning a specific prior to the variance components of the singular values. According to Cornelius et al. [26], and Cornelius and Crossa [25], the use of estimators with a shrinkage effect eliminates the model selection stage, but does not guarantee more parsimonious models with respect to the number of retained components for explaining the GEI. Oliveira et al. [27], in turn, implemented the shrinkage estimators for the singular values of the GGE model using the principle of maximum entropy to ascription priors (the authors called this model the Bayesian-GGE entropy model or just BGGEE). In the Bayesian analysis of the AMMI (or GGE) model the use of a prior that promotes shrinkage in in singular values predictors can offer more parsimonious model adjustment. For this, some model selection methods must be used to determine the optimal number of axes to be retained. As in multiplicative models the first principal components describe most of the variability, it is expected that few singular values are significant for describing the GEI pattern. On the other hand, the estimates of singular values related to the components with little importance to explain the GEI (those related to the largest dimensions) effect are shrunk to zero. This gives us a more plausible justification for using the AMMI-2 biplot (with the first two principal components) representation since the first axes are predicted in a more pronounced manner.

A difficulty faced in the use of the Bayes factor or model selection methods that use information criteria is the great demand for time and computational resources. This is due to the fact that all possible models, depending on the dimension, must be adjusted for comparison. From a Bayesian point of view, the uncertainty regarding the best model, which fits a given set of data, can simply be incorporated into the inference problem. Consequently, the model itself is considered as another unknown parameter to be estimated and, with that, each model has its probability distribution. In this context, approaches involving the construction of transdimensional Markov chains, which allow the alteration of the model’s dimension during the sampling process, have gained popularity [28].

Among the class of transdimensional methods, there is the Reversible Jump in Markov chain Monte Carlo (RJMCMC), initially proposed by Green [29]. This procedure can be seen as a generalization of the Metropolis-Hastings algorithm when the model’s parametric space does not have a fixed dimensionality [30]. This method allows for the flexibility to “jump” between models with parametric spaces of different dimensions, which ensure that the chain is irreducible. Consequently, from the output of a single Markov chain sampler, a complete probabilistic description of the posterior distributions of each model is obtained [28, 31, 32].

Therefore, the main objective of this study was to adjust the AMMI model under the Bayesian perspective, assuming that the number of principal axes to be retained in the model is a random variable. This assumption was implemented using the RJMCMC methodology for the parameter sampling process. Thus, the joint inference about the number of bilinear terms responsible for the GEI pattern is directly incorporated by the inclusion (or exclusion) of multiplicative terms in the model during the sampling process. Furthermore, specific objectives of the study were as follows: i) to evaluate the sensitivity of the AMMI-Bayesian model to the prior specification for the variance component associated with singular values; ii) use information criteria for model selection, and iii) an evaluation the predictive capacity of the model in a scenario of random loss of genotypes in environment.

To exemplify the use of the method in the evaluation of cultivars we will use simulated and real data. The real data comprise 50 maize genotypes evaluated in 10 locations in southern Brazil. We will address two points that are important in the final stages of plant breeding programs. The first will be to verify the existence of genotypes that have little contribution to the GEI (stable), but that are interesting in terms of yield (broad recommendation). The second will be to identify positive combinations between genotypes and environments, aiming to take advantage of the positive effect of the GEI (recommendation of genotypes to specific environments).

## Material and methods

### Simulated data

To exemplify the proposed method, a data set with 20 genotypes (G1-G20) and nine environments (E1-E9) was simulated, using a randomized block design, with three replicates. The main effects of genotypes (g) were sampled from a Gaussian distribution with mean equal to zero and variance equal to 12, *N*(0, 12), and the main effects of the environment sampled from a Gaussian distribution with mean equal to zero and variance equal to one *N*(0,1).

The effects of the GEI for each genotype by environment combination (*ge*_*ij*_) for *i* = 1,…,20 and j = 1,…,9, where *i* indicate the genotype and *j* indicate the environment, were sampled from Gaussian distribution, getting three subgroups (patterns) as highlighted below:

1st subgroup: Genotypes G1, G2, G3, G4, and G5 with positive effects in E1, E2, E3, E4 environments and negative effects in E5, E6, E7, E8, and E9 environments. The effects of *ge*_*ij*_ (*i* = 1,…,5) were sampled from a Gaussian distribution *N*(0, 16), and in the environments *j* = (1,…4) was considered as the module of the values and in the other environments *j* = (5,…9), the module of the values, was multiplied by (-1).
2nd subgroup: Genotypes G6, G7, G8, G9, and G10 with negative effects in E1, E2, E3, E4 environments and positive effects in E5, E6, E7, E8, and E9 environments. Similar to what was considered in the first subgroup, the effects of *ge*_*ij*_ (*i* = 6,…,10) were sampled from a Gaussian distribution *N*(0, 16). But for *j* = (1,…4) the values are negative, whereas for *j* = (5,…9) they are positive.
3rd subgroup: Genotypes containing G11, G12,… ., G20, whose GEI effects *ge*_*ij*_ (*i* = 11,…20), in the environments *j* = (1,…9), are sampled from a standard Gaussian distribution *N*(0,1).

The *ge*_*ij*_ effects thus simulated make up a *GE*_*g×e*_ matrix which is finally corrected for row and column mean so that it corresponds merely to the matrix genotype-by-environment interaction, where *g* represents the number of rows and *e* represents the number of columns.

Genotypes belonging to the 1st and 2nd groups were assumed to be unstable genotypes (contribute to interaction). By contrast, the genotypes belonging to the 3rd group were assumed as being stable (they do not have expressive contributions to interaction).

Thus, each value *y*_*ijk*_ was obtained by adding the simulated effects plus the error which, in turn, was simulated from a Gaussian distribution *N*(0,2.5). The simulation was performed using R software [33].

### Real data

The real data comprised 50 simple maize hybrids evaluated for grain yield character (t/ha^-1^) during the 2013 and 2014 crop years at 10 locations in the Southern Region of Brazil. The experiment was conducted in an incomplete block design with a variable number of repetitions.

#### Statistical model

The AMMI model, in vector notation, can be described as:

$$y=X_{1}\beta +Zg+\sum_{k=1}^{t}\lambda_{k}\;\text{diag}\left(Z\alpha_{k}\right)X_{2}\gamma_{k}+\epsilon$$ (1)

where ***y***_(*n*×1)_ is the vector of observations with *n* = *ger*, in which *g* corresponds to the number of genotypes, *e* to the number of environments, and *r* to the number of repetitions; **β**_(*er*×1)_ and ***g***_(*g*×1)_ are the vectors of block effects within environments and genotype effects, respectively; $\lambda_{k},\alpha_{k}{}_{\left(g\times 1\right)}$ and ${\gamma_{k}}_{\left(e\times 1\right)}$ correspond to, respectively, the singular value and the singular vectors (genotypic and environmental) associated with the *k*-th principal component, with *k* = 1, …,*t*, in which *t* is the rank of the GEI matrix, where the singular vectors satisfy the orthonormality constraints and the singular values satisfy the order constraint λ_*k*_ ≥ λ_*k*+1_ ≥ 0. The matrices ${X_{1}}_{\left(n\times er\right)},\;{X_{2}}_{\left(n\times e\right)}$ and, ***Z***_(*n*×*g*)_ are designs associated with effects **β**, **γ**_*k*_ and ***g***, respectively. As can be seen, ${X_{1}}_{\left(n\times er\right)}$ and ${X_{2}}_{\left(n\times e\right)}$ are different, where ${X_{2}}_{\left(n\times r\right)}$ is used to distribute the effects of the *GE*_(*g*×*e*)_ interaction and the number of columns is, therefore, equal to the number of environments. The ${X_{1}}_{\left(n\times er\right)}$ matrix, in turn, distributes the effects of blocks within environments, as in Silva et al. [24] and Edwards and Jannink [7] to maintain the identifiability of all parameters and not use marginal conditions where effects sum to zero as presented in Crossa et al. [11]. The vector *ϵ*_(*n*×1)_ is composed of random errors with $\epsilon \;\sim \;N\left(0,\sigma_{e}^{2}I_{n}\right)$.

If we denote by *GE* the interaction matrix, then it will have dimensions *g* × *e* (*g* row = genotype and *e* columns = environment). In the classic AMMI model, the mean of the cell is given by $y_{ij}=\mu +\beta_{i}+g_{j}+\sum_{k=1}^{t}\lambda_{k}\alpha_{ik}\gamma_{jk}+\epsilon_{ij}$, where now *α*_*ik*_ and *γ*_*jk*_ are coordinates of singular vectors of the *k*-th principal component. As our model is for parcels, the multiplications involving ***Z*** and ***X***_**2**_ in the sum of the model (1) allow distributing and replicating the effects of the GEI in the vector ***y***.

In model (1), the term *k* specifies the number of axes that are included in the model, which would be from *k* = 1, where only one axis is being incorporated into the model, up to *k* = *t* that comprises the complete model. However, in the approach presented here, the model space is *t* + 1, such that one can assume an index *k* = 0, where the corresponding model would be ***y*** = ***X***_1_**β** + ***Zg*** + ϵ, that is, the model disregarding the bilinear part.

The likelihood function is given by:

$$\text{L}(\bar{\theta }|y)={\left(2\pi \right)}^{-\frac{n}{2}}|I\sigma_{e}^{2}{|}^{-\frac{1}{2}}\;\text{exp}\left\{-\frac{1}{2\sigma_{e}^{2}}{\left(y-\theta \right)}^{⊤}\left(y-\theta \right)\right\}$$ (2)

where $\theta =X_{1}\beta +Zg+\sum_{k=1}^{t}\lambda_{k}diag\left(Z\alpha_{k}\right)X_{2}\gamma_{k}$ and $\overset{-}{\theta }=\left(\beta ,g,\lambda_{k},\alpha_{k},\gamma_{k},\sigma_{e}^{2}\right)$.

Prior distributions for the model parameters are as follows:

$$\beta |\mu_{\beta },\sigma_{\beta }^{2}∼\text{N}\left(\mu_{\beta }=0,\sigma_{\beta }^{2}\right),\;\text{with}\;\sigma_{\beta }^{2}=1\times 10^{8},$$

which implies

p(β)∝constant

$$g|\mu_{g},\sigma_{g}^{2}\sim N\left(0,I\sigma_{g}^{2}\right)$$

$$\lambda_{k}|\mu_{\lambda_{k}}\;\sigma_{\lambda_{k}}^{2}\;∼\;{\text{N}}^{+}\left(\mu_{\lambda_{k}}=0,\;\sigma_{\lambda_{\text{k}}}^{2}\right)$$

$$\alpha_{k}\sim \text{Uniform}\;\text{spherical}\;\text{distribution}\;\text{in}\;\text{the}\;\text{correct}\;\text{subspace}\;\left(SUNI_{d}\right)$$

$$\gamma_{k}\sim \text{Uniform}\;\text{spherical}\;\text{distribution}\;\text{in}\;\text{the}\;\text{correct}\;\text{subspace}\;\left(SUNI_{d}\right)$$

$$p(\sigma_{g}^{2})\propto \frac{1}{\sigma_{g}^{2}}$$

$$p(\sigma_{e}^{2})\propto \frac{1}{\sigma_{e}^{2}}$$

$$\text{t}∼p\text{oisson}-\text{truncated}\left(\mu \right)$$

$$\mu ∼g\text{ama}\left(\tau ,\upsilon \right)$$

For the singular value scale parameter, three hypotheses were established about the prior distribution:

**Hypothesis a**: It is assumed that $\sigma_{\lambda_{k}}^{2}=1\times 10^{8}$, which corresponds to assuming a non-informative prior for λ_*k*_. This is the hypothesis presented by Oliveira et al. [13], being referred to by Bayesian AMMI (BAMMI).
**Hypothesis b**: It is assumed that $\sigma_{\lambda_{k}}^{2}$ is a scaled inverted chi-square prior with scale parameter equal to zero, and degrees of freedom equal $\Delta =\frac{{(n}_{\lambda_{k}}-1)}{2}$ with the restriction of 0 < Δ < 1//2, resulting in a posterior with a shrinking effect for singular values. This is the Bayesian AMMI Shrinkage (BAMMIS) version proposed by Silva et al. [24]:
$$\sigma_{\lambda_{k}}^{2}∼E\text{sc}-\chi^{-2}\left(\upsilon_{\lambda_{k}},S_{\lambda_{k}}^{2}\right);p\left(\sigma_{\lambda_{k}}^{2}\right)\propto {\left(\sigma_{\lambda_{k}}^{2}\right)}^{\left(\Delta -1\right)}.$$
**Hypothesis c**: It is assumed that $\sigma_{\lambda_{k}}^{2}$ is an inverse gamma prior with degrees of freedom equal to one (*a* = 1) and scale parameter equal to zero (*b* = 0). This choice is based on the concept of Maximum Entropy (ME), and was implemented in the Bayesian GGE model by Oliveira et al. [27] (more details in S1 Appendix) as follows:
$$\sigma_{\lambda_{k}}^{2}∼I\text{nv}-gamma\left(a,b\right).$$

As specified, priors are non-informative for $\beta ,\;\sigma_{e}^{2}$, and genotypic and environmental singular vectors. However, for *g*, a hierarchical prior at two levels was assigned considering uncertainty about the hyperparameter $\sigma_{g}^{2}$, which results in considering a common population for genotypes. This is similar to what occurs in mixed models when genotype effects are considered random.

The prior distributions, assigned to the singular vectors, are uniform spherical in the corrected subspace due to the orthonormality constraint (the justification for such an assignment is elucidated in Viele and Srinivasan [9]). In this study, there is a special interest in the priors attributed to the singular values hyperparameters. The prior distribution of λ_*k*_ is a truncated normal for positive values, it is denoted by *N*^+^, with zero mean and with variance $\sigma_{\lambda_{k}}^{2}$, which was assumed in the three hypotheses about the prior that was utilized.

Furthermore, the number of principal axes (*t*) present in the model is a random variable whose a priori uncertainty was established through a truncated Poisson distribution with mean μ. For the μ hyperparameter, a Gamma distribution with scale parameter and degree of freedom equal to one was assigned, as assumed in Balestre and Souza [34].

#### Posterior complete conditional distributions for model parameters

Combining the likelihood function with the established prior distributions, using Bayes’ theorem, we obtain the joint posterior distribution.:

$$\text{P}\left(\Phi ,|y\right)\propto L\left(\phi ,\theta |y\right)\text{P}\left(\beta \right)\text{P}\left(g\right)\text{P}\left(\sigma_{g}^{2}\right)\text{P}\left(\sigma_{e}^{2}\right)\times \left\{\prod_{k=1}^{t}P\left(\lambda_{k}\right)P\left(\alpha_{k}\right)P\left(\gamma_{k}\right)\right\}\text{P}\left(t|\mu \right)\text{P}\left(\mu \right)$$ (3)

in which: $\Phi =\left(\beta ,g,\sigma_{g}^{2},\sigma_{e}^{2},\lambda_{k},\sigma_{\lambda_{k}}^{2},\alpha_{k},\gamma_{k},t,\mu \right)$.

The posterior complete conditional distributions are obtained from the joint distribution (3). They are presented, along with the algebraic details in the S2 Appendix.

### RJMCMC algorithm decision rule

The objective of using the RJMCMC algorithm was to conduct model selection during the MCMC process. This method allows one to switch between models with different dimensions such that the properties inherent in the MCMC process are not violated. As in this RJMCMC approach, the models are nested, and the decision rule is similar to the proposals of Waagepetersen and Sorensen [35], Bodin and Sambridge [36], and Balestre and Souza [34], where the Jacobian determinant of the transformation in the decision rule is equal to one.

Therefore, the decision rule to test the addition of a new main axis to the model is given by:

$$a\left(t,t+1\right)=\frac{\prod_{i=1}^{n}p\left(y|t+1\right)}{\prod_{i=1}^{n}p\left(y|t\right)}\frac{p\left(t+1|\mu \right)}{p\left(t|\mu \right)}\frac{\xi \left(t,t+1\right)}{\xi \left(t+1,t\right)}$$ (4)

where ***t*** corresponds to the current state model and *t* + 1 to the candidate model with the addition of an axis; *p*(*y*│*t*) and *p*(*y*│*t* + 1) are the probability functions of data from model *t* and (*t* + 1), respectively; *p*(*t*│μ) and *p*(*t* + 1│μ) are the priors referring to the respective models; ξ(*t*,*t* + 1) and ξ(*t* + 1,*t*) correspond to the proposed Hastings correction necessary to guarantee the reversibility condition of the model during the MCMC process, being $\xi \left(t,t+1\right)=\frac{1}{t+1}pd$ and ξ(*t* + 1,*t*) = *p*_0_ wherein *pa*, *pd* and *p*_0_ correspond to the prior probabilities of adding, deleting, or maintaining, respectively, the number of main axes in the model (*pa* = *pd* = *p*_0_ = 1/3).

Simplifying Eq (4), the decision rule for adding a dimension to the model is:

$$a\left(t,t+1\right)=\frac{\prod_{i=1}^{n}p\left(y|t+1\right)}{\prod_{i=1}^{n}p\left(y|t\right)}\frac{\mu }{t+1}\frac{pd}{\left(t+1\right)p_{0}}.$$

If *a*(*t*, *t* + 1) is greater than a random variable *u* ~ *U*(0,1), then the candidate axis is included in the model; otherwise, the model is retained with the current number of main axes present in the model *t*.

The next step in the algorithm is to carryout the test between the model with dimension *t* and the model with dimension *t* − 1. That is, the model with *t* principal components and the model with reduction of a principal component *t* − 1, where the decision rule is given by:

$$a\left(t,t-1\right)=\frac{\prod_{i=1}^{n}p\left(y|t-1\right)}{\prod_{i=1}^{n}p\left(y|t\right)}\;\frac{p\left(t-1|\mu \right)}{p\left(t|\mu \right)}\;\frac{\xi \left(t,t-1\right)}{\xi \left(t-1,t\right)}$$ (5)

Simplifying Eq (5), the decision rule to accept or not accept the exclusion of a dimension in the model is:

$$a\left(t,t-1\right)=\frac{\prod_{i=1}^{n}p\left(y|t\right)}{\prod_{i=1}^{n}p\left(y|t-1\right)}\frac{t}{\mu }\frac{pa\;t}{p_{0}}$$

where *p*(*y*|*t* − 1) is the conditional distribution of data for the dimension-reduced model (*t* − 1) and with *ξ*(*t*, *t* − 1) and *ξ*(*t* − 1, *t*) corresponding to the necessary Hastings corrections to guarantee the reversibility conditions in the sampling, with *ξ*(*t*, *t* − 1) = *p*_0_ and *ξ*(*t* − 1, *t*) = *pd*/*t*.

### Sampling process

The sampling of the parameters of the models was performed iteratively, combining the Gibbs sampler and the RJMCMC method. The Gibbs algorithm was arranged as follows:

#### Gibbs algorithm

A1—Initial values are assigned to model parameters:

$$\left(\beta^{0},g^{0},\lambda_{k}{}^{0},\alpha_{k}{}^{0},\gamma_{k}{}^{0},{\left(\sigma_{\lambda_{k}}^{2}\right)}^{0},{\left(\sigma_{g}^{2}\right)}^{0},{\left(\sigma_{e}^{2}\right)}^{0},\mu^{0}\right).$$

A2—The *l*-th iteration can be obtained as follows:

- Generates $\beta^{l}|g^{l-1},{\lambda_{k}}^{l-1},{\left(\sigma_{\lambda_{k}}^{2}\right)}^{l-1},{\alpha_{k}}^{l-1},{\gamma_{k}}^{l-1},{\left(\sigma_{g}^{2}\right)}^{l-1},{\left(\sigma_{e}^{2}\right)}^{l-1},\mu^{l-1}$ from the conditional posterior distribution:
$$\beta |\cdots ∼N\left[{\left(X_{1}^{⊤}X_{1}\right)}^{-1}X_{1}^{⊤}A_{1};{\left(X_{1}^{⊤}X_{1}\right)}^{-1}\sigma_{e}^{2}\right],$$
  where: $A_{1}=y-Zg–\sum_{k=1}^{t}\lambda_{k}diag\left(Z\alpha_{k}\right)X_{2}\gamma_{k}$.
- Generates $g^{l}|\beta^{l},{\lambda_{k}}^{l-1},{\left(\sigma_{\lambda_{k}}^{2}\right)}^{l-1},{\alpha_{k}}^{l-1},{\gamma_{k}}^{l-1},{\left(\sigma_{g}^{2}\right)}^{l-1},{\left(\sigma_{e}^{2}\right)}^{l-1},\mu^{l-1}$ from the conditional posterior distribution:
$$g|\cdots ∼N\left[{\left(Z^{⊤}Z+I\sigma_{g}^{2}\right)}^{-1}Z^{⊤}A_{2};{\left(Z^{⊤Z}+I\sigma_{g}^{2}\right)}^{-1}\sigma_{e}^{2}\right],$$
  where: $A_{2}=y-X_{2}\beta –\sum_{k=1}^{t}\lambda_{k}diag\left(Z\alpha_{k}\right)X_{2}\gamma_{k}.$
- Generates ${\lambda_{k}}^{l}|\beta^{l},g^{l},{\left(\sigma_{\lambda_{k}}^{2}\right)}^{l-1},{\alpha_{k}}^{l-1},{\gamma_{k}}^{l-1},{\left(\sigma_{g}^{2}\right)}^{l-1},{\left(\sigma_{e}^{2}\right)}^{l-1},\mu^{l-1}$ from the conditional posterior distribution:
$$\lambda_{k}|\cdots ∼N^{+}\left[{\left(\phi_{k}^{⊤}\phi_{k}+\frac{\sigma_{e}^{2}}{\sigma_{\lambda_{k}}^{2}}\right)}^{-1}\phi_{k}^{⊤}A_{4k};{\left(\phi_{k}^{⊤}\phi_{k}+\frac{\sigma_{e}^{2}}{\sigma_{\lambda_{k}}^{2}}\right)}^{-1}\sigma_{e}^{2},b=\lambda_{k-1}\right],$$
  where $A_{4k}=y-X_{1}\beta -Zg-\sum_{k'\neq k}^{t-1}\lambda_{k}diag\left(Z\alpha_{k'}\right)X_{2}\gamma_{k'},$ ***ϕ***_k_ = *diag*(***Z*α**_***k***_)***X***_2_**γ**_*k*_, and *λ*_0_ = +∞.
  Step “D” refers to the component of variance related to the singular value for which three hypotheses are specified. Here, only hypothesis “c” will be used to illustrate the sampling process. For the other hypotheses, the process is analogous.
- Generates ${\left(\sigma_{\lambda_{k}}^{2}\right)}^{l}|\beta^{l},g^{l},{\lambda_{k}}^{l},{\alpha_{k}}^{l-1},{\gamma_{k}}^{l-1},{\left(\sigma_{g}^{2}\right)}^{l-1},{\left(\sigma_{e}^{2}\right)}^{l-1},\mu^{l-1}$ from the conditional posterior distribution:
$$\sigma_{\lambda_{k}}^{2}|\ldots \sim Inv-Gamma\left(\frac{2\upsilon_{\lambda_{k}}+1}{2};\frac{\left[S_{\lambda_{k}}+\lambda_{k}\right]}{2}\right).$$
- Generates ${\alpha_{k}}^{l}|\beta^{l},g^{l},{\lambda_{k}}^{l},{\left(\sigma_{\lambda_{k}}^{2}\right)}^{l},{\gamma_{k}}^{l-1},{\left(\sigma_{g}^{2}\right)}^{l-1},{\left(\sigma_{e}^{2}\right)}^{l-1},\mu^{l-1}$ from the conditional posterior distribution:
$$\alpha_{k}|.\;.\;.\sim VMF\left(\frac{\lambda_{k}}{\sigma_{e}^{2}};\Delta_{1k}^{⊤}\left(y-X_{1}\beta -Zg\right)\right),$$
  where: **Δ**_1*k*_ = *diag*(***X***_2_γ_k_)***Z***
- Generates ${\gamma_{k}}^{l}|\beta^{l},g^{l},{\lambda_{k}}^{l},{\left(\sigma_{\lambda_{k}}^{2}\right)}^{l},{\alpha_{k}}^{l},{\left(\sigma_{g}^{2}\right)}^{l-1},{\left(\sigma_{e}^{2}\right)}^{l-1},\mu^{l-1}$ from the conditional posterior distribution:
$$\gamma_{k}|\ldots \sim VMF\left(\frac{\lambda_{k}}{\sigma_{e}^{2}};\Delta_{2}^{⊤}\left(y-X_{1}\beta -Zg\right)\right),$$
  where: **Δ**_2*k*_ = *diag*(***Z***α_k_)***X***_2_.
- Generates ${\left(\sigma_{g}^{2}\right)}^{l}|\beta^{l},g^{l},\lambda_{k}^{l},{\left(\sigma_{\lambda_{k}}^{2}\right)}^{l},\alpha_{k}^{l},\gamma_{k}^{l},{\left(\sigma_{e}^{2}\right)}^{l-1},\mu^{l-1}$ from the conditional posterior distribution:
$$\sigma_{g}^{2}|.\;.\;.\sim Esc-\chi^{-2}\left[n_{g};\frac{g^{⊤}I_{g}g}{n_{g}}\right].$$
- Generates ${\left(\sigma_{e}^{2}\right)}^{l-1}|\beta^{l},g^{l},\lambda_{k}^{l},{\left(\sigma_{\lambda_{k}}^{2}\right)}^{l},\alpha_{k}^{l},\gamma_{k}^{l},{\left(\sigma_{g}^{2}\right)}^{l},\mu^{l-1}$ from the conditional posterior distribution:
$$\sigma_{e}^{2}|.\;.\;.\sim Esc-\chi^{-2}\left[n_{e};\frac{\left(y-\theta {)}^{⊤}(y-\theta \right)}{n_{e}}\right].$$
- Generates $\mu^{l}|\beta^{l},g^{l},\lambda_{t}^{l},\alpha_{t}^{l},\gamma_{t}^{l},{\left(\sigma_{\lambda_{t}}^{2}\right)}^{l},{\left(\sigma_{g}^{2}\right)}^{l},{\left(\sigma_{e}^{2}\right)}^{l}$ from the conditional posterior distribution:
$$\mu |\cdots ∼Gamma\left(\tau ,\mu \right).$$

After carrying out the sampling process parameters of the model (*t*) in the *l*-th iteration, the additional parameters referring to the concurrent model (*t* + 1) are sampled.

The size of the model is given by the number of multiplicative terms retained to explain GEI (0, 1, 2,…, *rank*(GEI)). For each proposed model, this dimension is directly related to steps C, D, E, and F of the Gibbs sampling process. For the model without the presence of the multiplicative part *t* = 0, steps C, D, E, and F are not included in the sampling.

#### RJMCMC algorithm

With the sampling process for the parameters of the candidate models already established, the next step is to test the models, using the decision rule, selecting the most probable. For this, the following steps of the algorithm are performed:

- Inclusion movement
  1. Propose the addition of a bilinear term in the model (*t* + 1);
  2. Calculate the probability of accepting *a*(*t*, *t* + 1), the inclusion of the bilinear term;
  3. Sampling a random number from a uniform distribution *u* ~ *U*(0,1);
  4. If *u* < *min*(1, *a*(*t*, *t* + 1)), the proposed state is accepted; otherwise, the model is retained with the initial dimension.
- Exclusion movement:
  1. Propose the removal of a bilinear term in the model (*t* − 1);
  2. Calculate the probability of accepting *a*(*t*, *t* − 1) the exclusion of the bilinear term;
  3. Sampling a random number from a uniform distribution *u* ~ (0,1);
  4. If *u* < *min*(1, *a*(*t*, *t* − 1)), the proposed state is accepted; otherwise, the model remains with the initial dimension.

Repeat the steps specified in i) and ii) during the iterative process.

The model selected to represent the dataset will be the one with the highest frequency of visits, based on the decision rule.

### The predictive capacity of the model

To assess the predictive capacity of the models with and without the use of the RJMCMC algorithm, an unbalance process was performed on the data such that within each environment, random losses of genotypes were established (total loss of the genotype) at the level of loss of 10% of the data set. Thus, the original sample was randomly divided into 10 sub-samples (10-fold), keeping all the genotype and environment information in the sub-samples. In each fold, the data were divided into training and validation data.

The predictive capacity of the models was evaluated using the PRESS (Predicted Residual Sum of Squares) criteria and the correlation (Cor) between the predicted (${\hat{y}}_{ijr}$) and observed (*y*_*ijr*_) phenotypic values. This verification was given in scenarios involving the different hypotheses of the variance component related to the singular value.

Cor and PRESS are calculated, respectively, by:

$$Cor=\frac{\sum_{i=1}^{n}\left({\hat{y}}_{ijr}-\;\;{\overset{-}{\hat{y}}}_{ijr}\right)\left(y_{ijr}-{\overset{-}{y}}_{ijr}\right)}{\sqrt{\sum_{i=1}^{n}{\left({\hat{y}}_{ijr}-\;\;{\overset{-}{\hat{y}}}_{ijr}\right)}^{2}\sqrt{\sum_{i=1}^{n}{\left(y_{ijr}-{\overset{-}{y}}_{ijr}\right)}^{2}}}}$$

and

$$PRESS=\frac{1}{n}\sum_{i=1}^{n}{\left(y_{ijr}-{\hat{y}}_{ijr}\right)}^{2}$$

where ${\overset{-}{\hat{y}}}_{ijr}$ is the predicted mean values, ${\overset{-}{y}}_{ijr}$ the mean of the observed values and *n* denotes the number of data removed for validation.

### Diagnosis of Markov chains and a posteriori inference

The total number of iterations to be performed with the Gibbs sampler in each analysis, was determined by a pilot sample according to Raftery and Lewis [37], indicating the number of initial observations to be discarded (burning), and the jumps necessary to collect the observations (tinning) that will compose the sample for inference. These procedures were performed to avoid selecting values from non-stationary strings and/or autocorrelated strings.

The convergence of the produced Markov chains, after the correction procedures described above, was monitored using the method of Raftery and Lewis [37] and by the criterion of Heidelberger and Welch [38]. Trace and density plots were used for visual analysis of the stationarity of Markov chains.

The singular vectors, in turn, are estimated from a correction to preserve the orthonormality constraints between the vectors [10]. This is done by orthonormalizing the matrices "**U**" and "**V**", whose columns are composed by the average coordinates of the genotypic and environmental singular vectors, respectively.

Linear parameters, singular values, and variance components of the selected model were estimated by posterior means of the simulated distributions. In addition, uncertainty regarding parameter estimation was quantified through the highest posterior density credible region (HPD).

Posterior bivariate credibility regions for genotypic and environmental scores were incorporated into the biplot-AMMI using the method described by Hu and Yang [39] to study stability and adaptability.

For models adjusted by the RJMCMC method, posterior means, HPD regions with 95% credibility, and bivariate credibility regions for genotypic and environmental scores in the biplot were obtained from two perspectives:

- Based on the most likely model, which is the one that considers only the observations contained in the conditional model (the model with the highest frequency of “visits”), which is referred to as conditional response herewith.
- Based on the marginal model that considers all MCMC observations of the possible models that contain the estimated parameters present in the most likely model; that is, it considers the estimates of nested models in which the number of bilinear terms is equal to or greater than that of the most likely model. This is referred to in the text as marginal response.

The entire inference process, as well as the convergence tests, were conducted using the boa package [40] and other tools from the R statistical software.

## Results

The results obtained by applying the criteria of Heidelberger and Welch [38], and Raftery and Lewis [41] indicated good convergence properties for MCMC chains (Gibbs sampling) for all parameters with a dependence factor (I) always lower than five (I<5). Furthermore, all parameters passed the stationarity test, indicating that convergence was achieved.

### Model selection using information criteria

**Fig 1:**
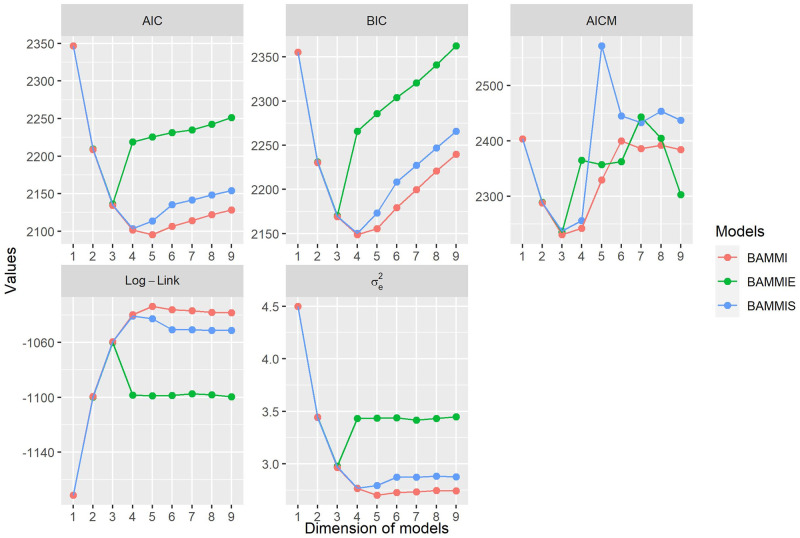
Ockham’s plot for the BAMMI, BAMMIS, and BAMMIE models for the AIC, AICM, and BIC information criteria, as well as for the log-likelihood (log-like) and residual variance (*σ*_*e*_^2^).

Based on Fig 1, the model with the best fit, according to the AICM criterion, is independent of the assumed prior for the variance components of the singular value, being the one with two retained bilinear terms (AMMI-2). For BAMMIE, the model that best fits the data was also the same (AMMI-2), regardless of the selection criteria used. For BAMMI and BAMMIS, the best fit models were, respectively, AMMI-3 for the BIC; for the AIC, it was AMMI-4 and AMMI-3, for BAMMI and BAMMIS, respectively.

Fig 1 also presents the description of the behavior of the models concerning the log-likelihood (Log-Link) and residual variance (*σ*_*e*_^2^). It is noteworthy that this information, by itself, does not correspond to tests to determine the number of bilinear components that should be retained in the model, but is useful for describing the behavior of the data and can still be used as auxiliary criteria in the step of selection.

Table 1 presents posterior means of the singular values for the models as a function of the number of retained bilinear terms, as well as the respective sum of squares of the accumulated GEI to each dimension. An accentuated shrinkage effect was observed in the estimates of the singular values of BAMMIE, concerning the other models, from the adjustment of the third multiplicative term. This accentuated shrinkage was also seen in BAMMIS concerning BAMMI, as from the model with a three-axis. S1 Table presents “true” values for the λ_*k*_, together with the respective GEI sums of squares, conditioned to each dimension of the model, and were obtained by Singular Value Decomposition (SVD) of the simulated interaction matrix, in which the sum of squares accumulated was 259.6064.

**Table 1:** Posterior means of singular values referent of each model (AMMI) and their respective accumulated sum of squares of the interaction in each dimension (Gibbs algorithm).

BAMMI									
Dim.	*λ*_1_	*λ*_2_	*λ*_3_	*λ*_4_	*λ*_5_	*λ*_6_	*λ*_7_	*λ*_8_	SS(GEI)
1	12.97								168.24
2	13.13	8.63							246.84
3	13.17	8.22	6.12						278.36
4	13.2	8.25	5.38	3.83					285.96
5	13.21	8.22	5.37	2.66	1.77				281.07
6	13.21	8.25	5.37	2.67	1.24	0.75			280.51
7	13.19	8.22	5.32	2.64	1.23	0.59	0.33		278.73
8	13.19	8.22	5.33	2.63	1.23	0.57	0.27	0.14	278.79
BAMMIS									
	*λ*_1_	*λ*_2_	*λ*_3_	*λ*_4_	*λ*_5_	*λ*_6_	*λ*_7_	*λ*_8_	SS(GEI)
1	12.87								165.7
2	13.05	8.5							242.76
3	13.1	8.08	5.91						271.89
4	13.09	8.09	4.18	2.45					260.49
5	13.07	7.98	3.85	0.56	0.11				249.62
6	13.08	7.99	3.87	0.55	0.05	0.01			250.31
7	13.08	7.99	3.78	0.55	0.05	0.001	<0.001		249.39
8	13.07	7.99	3.83	0.52	0.05	0.001	<0.001	≪0.001	249.66
BAMMIE									
	*λ*_1_	*λ*_2_	*λ*_3_	*λ*_4_	*λ*_5_	*λ*_6_	*λ*_7_	*λ*_8_	SS(GEI)
1	12.65								160.09
2	12.87	8.23							233.31
3	12.68	0.27	0.001						160.9
4	12.66	0.23	0.001	<0.001					160.29
5	12.62	0.27	0.001	<0.001	<0.001				159.34
6	12.67	0.55	0.002	<0.001	<0.001	<0.001			160.73
7	12.65	0.39	0.001	<0.001	<0.001	<0.001	≪0.001		160.17
8	12.65	0.05	0.001	<0.001	<0.001	<0.001	≪0.001	≪0.001	160.15

Dim., model dimension; SS(GEI), sum of squares for GEI; A≪B implies A<0.01B.

A point to be noted is that no member of the BAMMIE family overestimated the total variance of the GEI (simulated), and its estimates were always smaller concerning BAMMIS and BAMMI. Combining information from Fig 1 and Table 1, it is observed that models selected by the AICM criterion did not present an accumulated sum of squares greater than the true total sum of squares. This fact was also verified for BAMMIE, regardless of the criteria. For the models selected in BAMMIS and BAMMI using AIC and BIC, the sum of squared estimates retrieved was greater than the total simulated sum of squares.

Another finding was that the BAMMIE model selected by all information criteria (AMMI-2) presented a less discrepant accumulated sum concerning the first two terms of the SVD solution (S1 Table) compared to the others. However, considering complete models, BAMMIE was the one that most underestimated the true total sum of squares, BAMMI was the one that most overestimated it, and BAMMIS offered the closest value.

Summaries with a posteriori estimates of model parameters, including credibility regions for genotype effects and biplot representations, with incorporated bivariate credibility regions for genotypic and environmental scores that describe the GEI, referring to the adjustment using only Gibbs sampling, are presented in S1–S6 Figs and S2 Table.

### Adjustment of the AMMI model using the Reversible Jump method

**Fig 2:**
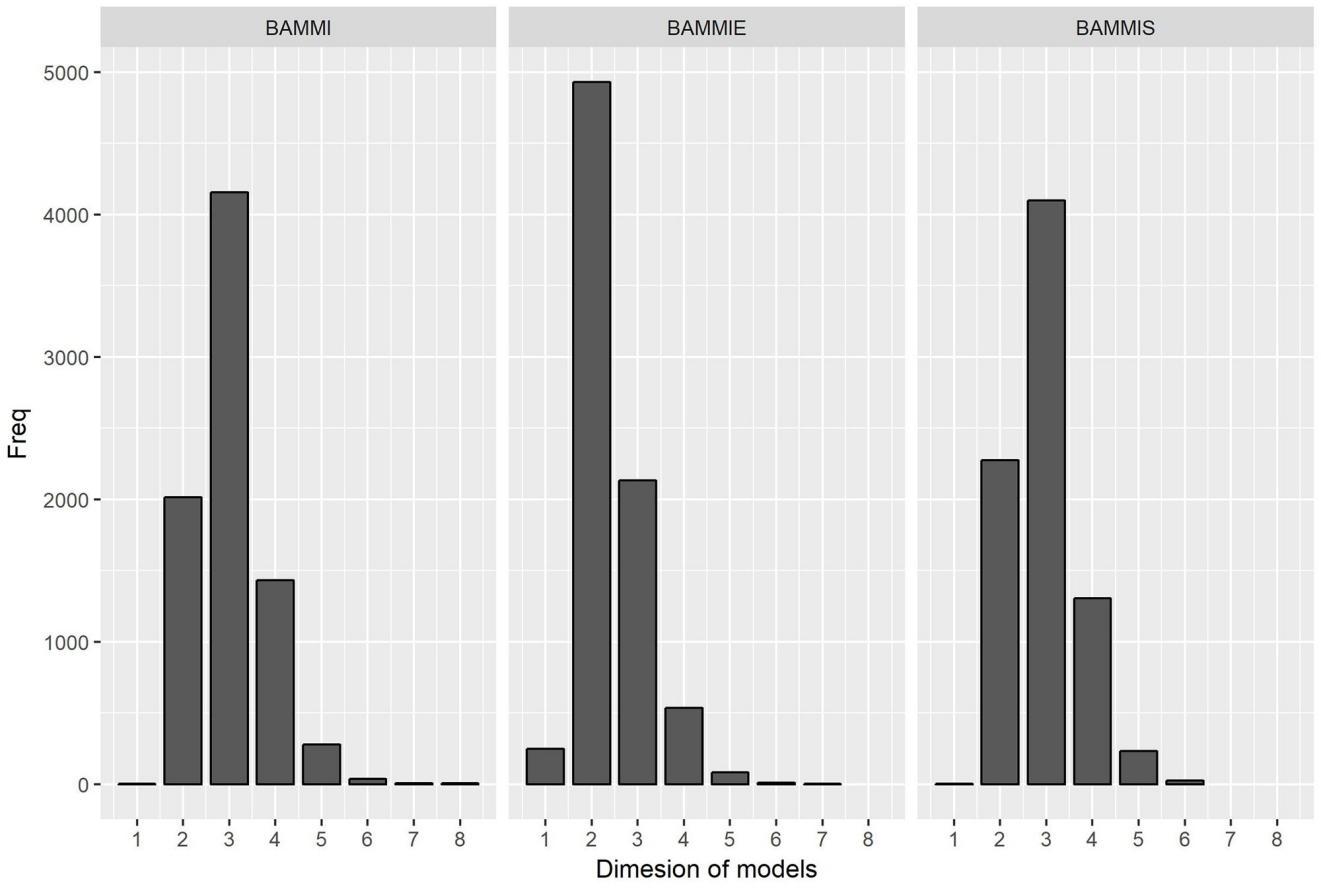
Frequency referring to the dimension (t) of the model accepted during the RJMCMC (simulated data).

Fig 2 illustrates the frequencies at which each model was visited in the RJMCMC sampling. These frequencies were used as criteria to select the winning model (most likely). According to this criterion, models AMMI3, AMMI2, and AMMI3 respectively would be selected for BAMMI, BAMMIE, and BAMMIS. Note that there are significant differences between the frequency of the winning model concerning the others for each AMMI version.

Table 2 indicates that the models presented very close estimates for the mean of the first two singular values. Furthermore, BAMMI and BAMMIS obtained similar mean values concerning λ3. Also, in relation to *λ*_3_, there is a more expressive difference for BAMMIE, although the HPD intervals overlap with 95% probability.

**Table 2:** Estimated singular values for the AMMI model referring to the three hypotheses made under the interaction, using the RJMCMC algorithm.

Model	Singular Value	Mean	SD	LL	UL
BAMMI	*λ*_1_	13.1993	1.0088	11.3004	15.2613
	*λ*_2_	8.2067	1.0520	6.1417	10.2358
	*λ*_3_	5.5862	1.1914	3.2555	7.8862
BAMMIE	*λ*_1_	12.8496	1.0481	10.8374	14.9333
	*λ*_2_	7.2263	2.0593	5.2887	9.8850
	*λ*_3_	0.4213	1.3676	*<* 0.001	4.5682
BAMMIS	*λ*_1_	13.0974	1.0059	11.0445	15.0142
	*λ*_2_	8.0646	1.1067	5.8534	10.2091
	*λ*_3_	5.2633	1.4088	2.4312	7.7224

SD, standard deviation; LL, lower limit; UL, upper limit

The sum of squares for the selected models BAMMI, BAMMIE, and BAMMIS were 272.7710, 217.3316, and 264.2804, respectively. Thus, the most probable models for BAMMI and BAMMIS overestimated the GEI total sum of squares, while BAMMIE had a relatively smaller value and was closer to the true sum of squares than that obtained by the AICM criterion (S1 Table). The accentuated shrinkage effect for the posterior mean of λ3 in the BAMMIE model, in relation to the others, as well as in relation to the real value (S1 Table), is remarkable, which indicates that the estimates for the other parameters should be shrunk to zero. This sharp shrinkage effect was not observed in the other selected models.

Posterior means and HPD credibility regions for residual and genotypic variances for the conditional and marginal responses of the models are presented in Table 3. No large discrepancies were observed between the conditional and marginal estimates or between the three models. Even when compared with the estimates of the models selected by the information criteria, it was found that there are no major discrepancies about genotypic and residual variances (S2 Table).

**Table 3:** Posterior means the standard deviation (SD) and the 95% HPD intervals (LL: lower limit, LU: upper limit) for components of variance genotypic (*σ*_*g*_^2^) and residual (*σ*_*e*_^2^) for models with conditional and response responses.

Model		Par.	mean	SD	LL	UL
BAMMI	Marginal Conditional	*σ*_*g*_2	11.973	4.330	5.258	20.228
		*σ*_*e*_2	2.841	0.209	2.454	3.257
		*σ*_*g*_2	12.028	4.381	5.367	20.391
		*σ*_*e*_2	2.810	0.182	2.464	3.161
BAMMIS	Marginal Conditional	*σ*_*g*_2	12.026	4.386	5.205	20.626
		*σ*_*e*_2	2.869	0.210	2.470	3.277
		*σ*_*g*_2	12.043	4.525	5.205	20.980
		*σ*_*e*_2	2.834	0.187	2.473	3.188
BAMMIE	Marginal Conditional	*σ*_*g*_2	12.028	4.514	5.181	20.735
		*σ*_*e*_2	3.054	0.223	2.658	3.530
		*σ*_*g*_2	12.032	4.567	5.181	20.783
		*σ*_*e*_2	3.050	0.209	2.676	3.486

Par., parameter

**Fig 3:**
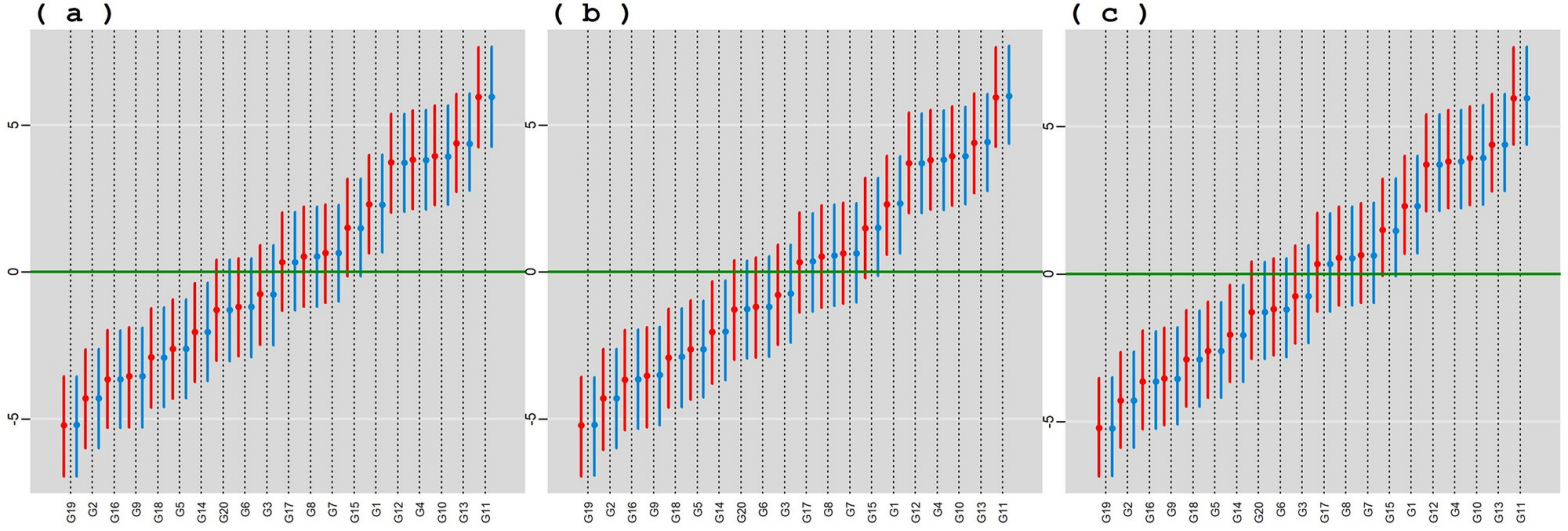
Posterior means and the 95% HPD intervals for the effect of genotypes, conditional response (blue color) and marginal responses (red color) referring to: a) BAMMI-3; b) BAMMIS-3; c) BAMMIE-2.

In Fig 3, posterior means and HPD intervals at 95% of credibility of the genotypic effects for BAMMI, BAMMIS, and BAMMIE are presented considering conditional (blue color) and marginal (red color) responses. In both situations, the pattern in the classification of main genotypic effects was practically the same between the models. Overlap between ranges indicates similar effects.

Regarding yield, the same subgroup of genotypes (more productive) {G1, G12, G4, G10, G13, G11} was selected for the three models. The subgroup {G19, G2, G16, G9, G18, G5, G14} had a significantly lower mean than the overall one (negative effect), not being interesting in terms of productivity. Lastly, there are those genotypes whose HPD regions include the origin and, therefore, the effects do not differ significantly from zero. Differences between the main effect estimates between the models are practically imperceptible graphically. These results are presented in S3–S5 Tables.

### Biplot credibility regions for models adjusted by Reversible Jump

**Fig 4:**
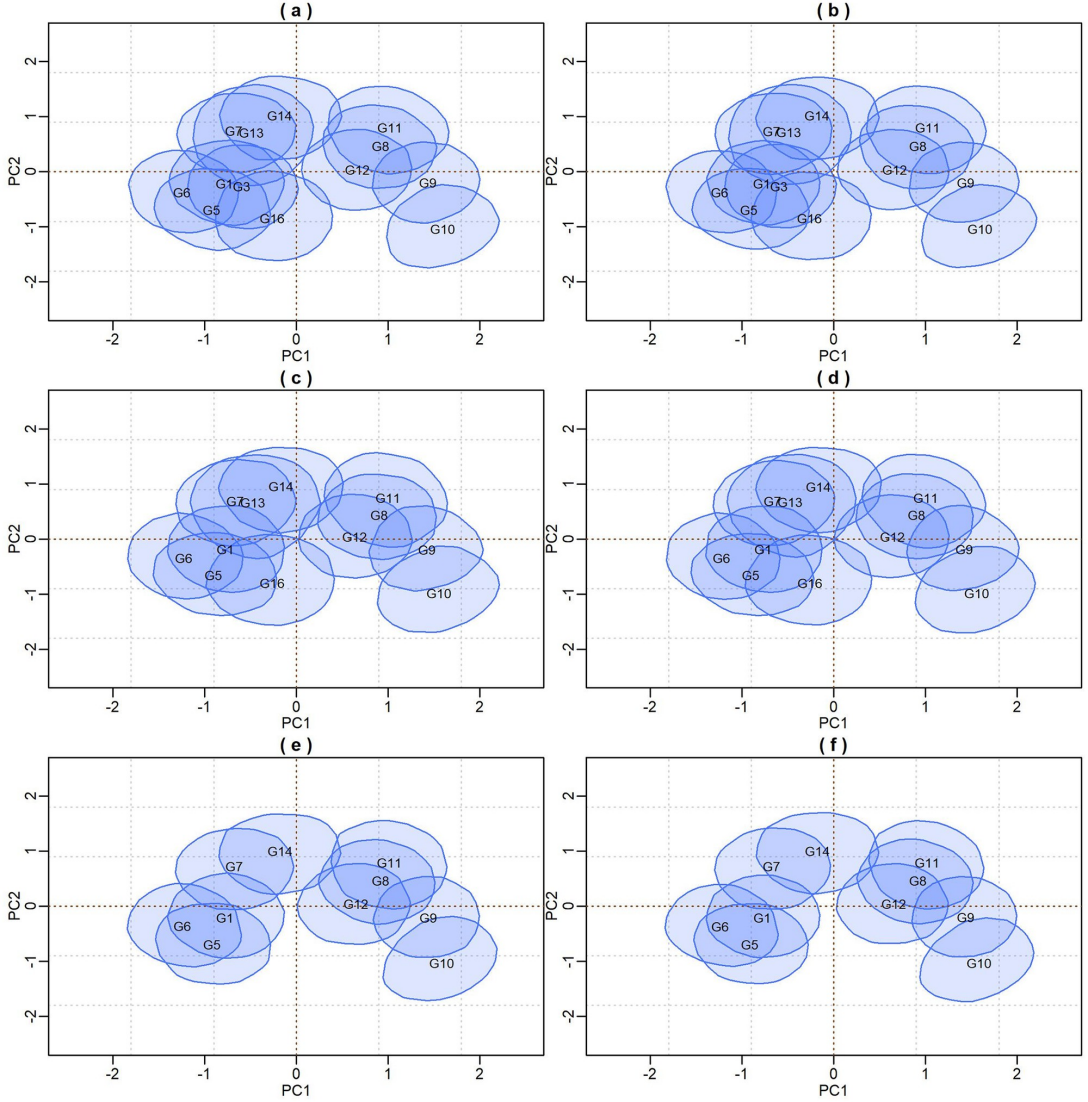
Representations of the bivariate credibility regions at 95% probability, based on the marginal and conditional response of the BAMMI, BAMMIS, and BAMMIE, models.

In Fig 4, biplots with posterior credibility regions for the genotypic scores of the three models are presented, with two representations for each model: marginal response and conditional response (as explained earlier in the Methods). From the visualization of the biplots, it is clear (for each model) that the two representations (marginal or conditional) did not result in differences in interpretation. However, there is clearly a change in the stability rating when comparing different models. As the responses within each model did not differ, conclusions about existing GEI standards will be made based on the marginal response.

**Fig 5:**
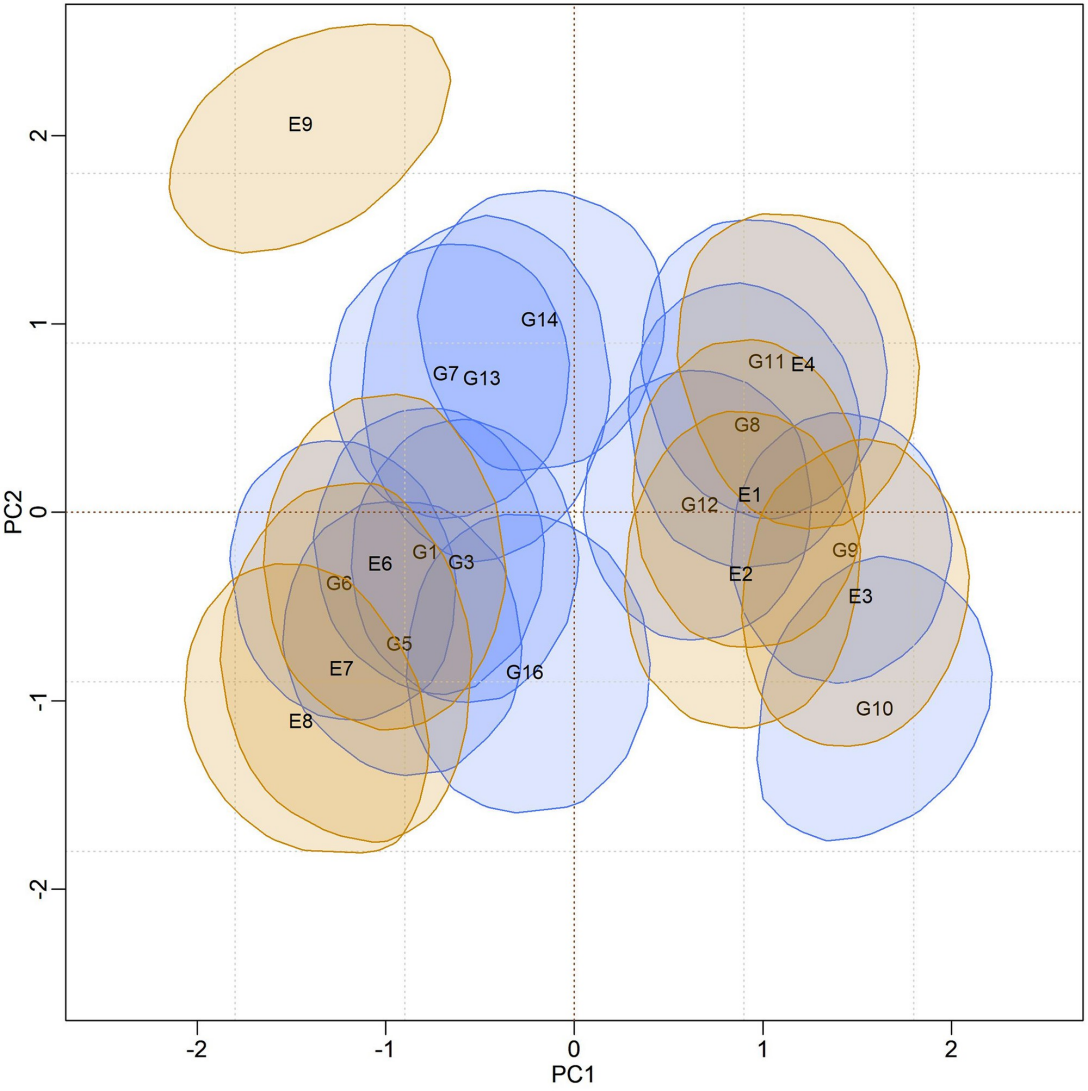
Bivariate credibility regions at 95% probability for the genotypic and environmental scores of the BAMMI model whose regions do not contain the origin (0.0).

Fig 5 presents the biplot with the credibility regions for genotypic and environmental scores (at 95% probability) referent the marginal BAMMI model. Regions that do encompass the origin (0,0) are not plotted for ease of interpretation biplot. The subgroup of genotypes formed by {G2, G4, G15, G17, G18, G19, G20}, which were not represented, were interpreted as stable, as they have no important contribution to the GEI. The same interpretation applies to the environments and, in this sense, E5, which was not represented, was considered stable. The subgroups of genotypes and environments, whose associated bivariate regions do not encompass the origin, effectively contribute to the GEI effect and are often referred to as unstable.

Furthermore, it is possible to identify similar subgroups of genotypes and environments concerning the interaction effect (homogeneous subgroups). Although with some overlaps that make it difficult to observe separable groups, one could identify five homogeneous subgroups of genotypes {G1, G3, G5, G6, G16}, {G8, G11, G12}, {G9, G10}, {G7, G13, G14} and {G2, G4, G15, G17, G18, G19, G20}, the latter being formed by stable genotypes. For environments, four homogeneous subgroups could be suggested, namely: {E6, E7, E8}, {E1, E2, E3, E4}, {E9} and {E5}. The adaptability of genotypes to specific environments can also be suggested from the biplot representation observing regions in the same quadrant and/or that have expressive overlaps.

**Fig 6:**
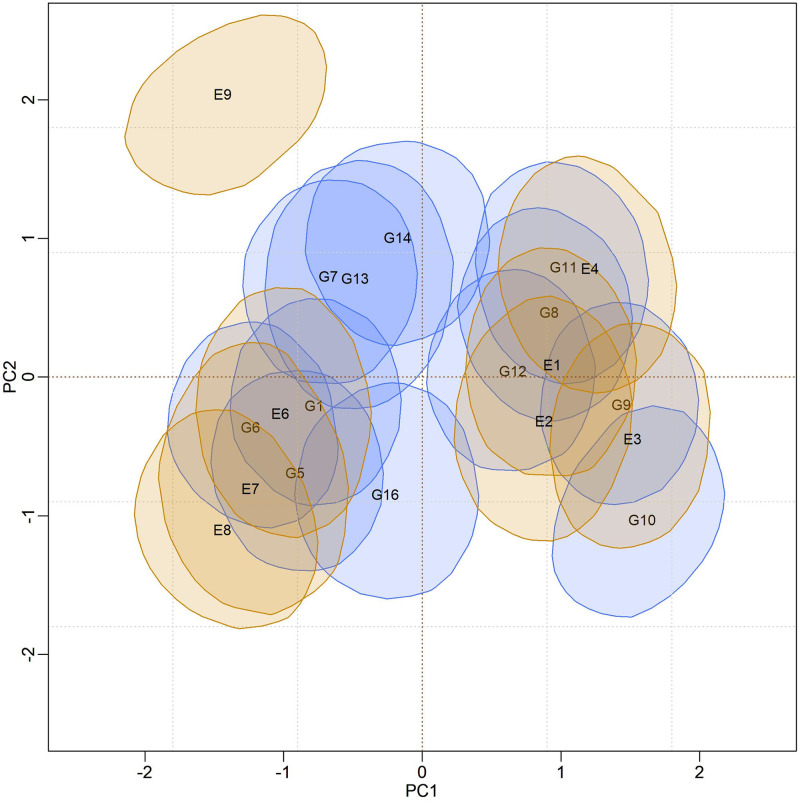
Bivariate credibility regions at 95% probability for the genotypic and environmental scores of the BAMMIS model whose regions do not contain the origin (0.0).

**Fig 7:**
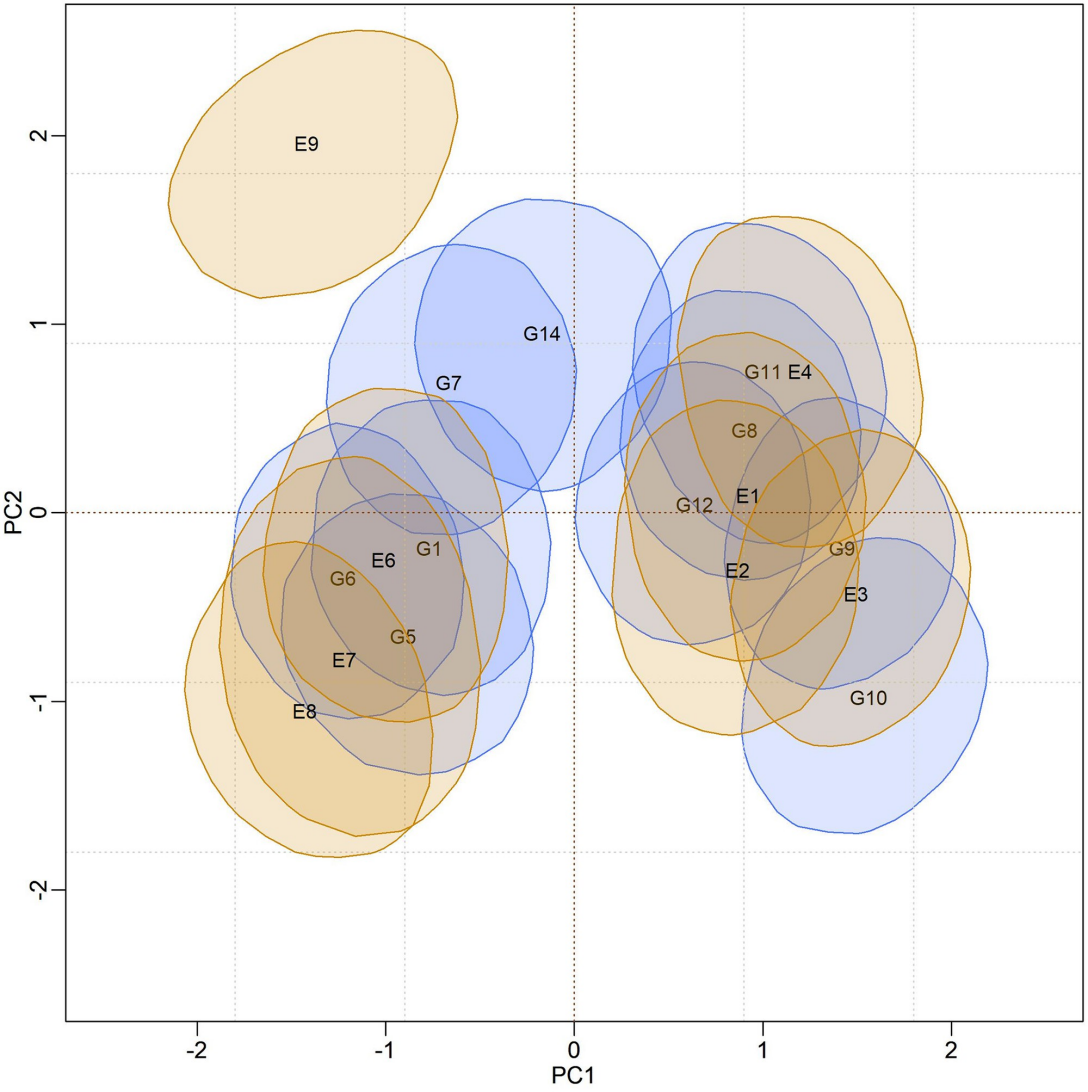
Bivariate credibility regions at 95% probability for the genotypic and environmental scores of the BAMMIE model whose regions do not contain the origin (0.0).

The biplots for the BAMMIS and BAMMIE models are shown in Figs 6 and 7, respectively. The only difference between BAMMI and BAMMIS is the G3 genotype, which was classified as stable in the BAMMIS analysis. In the BAMMIE biplot, the credibility regions of G3 and G16 also encompass the origin, signaling that these genotypes would not be important for the effect of GEI, which are the main differences in interpretations of BAMMIE concerning BAMMI and BAMMIS.

### Evaluation of the predictive capacity of the models

The evaluation of the predictive capacity of the models utilizing the Cor (the higher the value, the better) and PRESS (smaller values indicate a greater predictive capacity) statistics, was performed using the Gibbs and RJMCMC (marginal responses of the models) algorithms.

**Fig 8:**
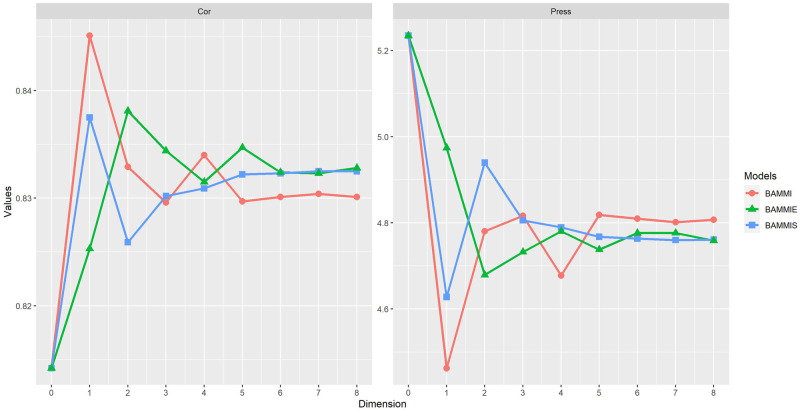
Ockham’s plot for the mean evaluation of the predictive capacity of the models based on the criteria of correlation between the observed and estimated values and the PRESS (Gibbs algorithm).

Fig 8 illustrates the behaviors of the three AMMI versions adjusted by the Gibbs sampler, based on mean Cor and PRESS. The results suggest a high predictive capacity for the models under the used imbalance (always above 81%), with emphasis on the BAMMI-1 that has a correlation above 84%. For the PRESS criterion, the models that presented the best results were BAMMI-1, BAMMIS-1, and BAMMIE-2, similar to the results obtained based on the correlation; note that the BAMMI-1 model achieved slightly higher results in both evaluation criteria.

**Fig 9:**
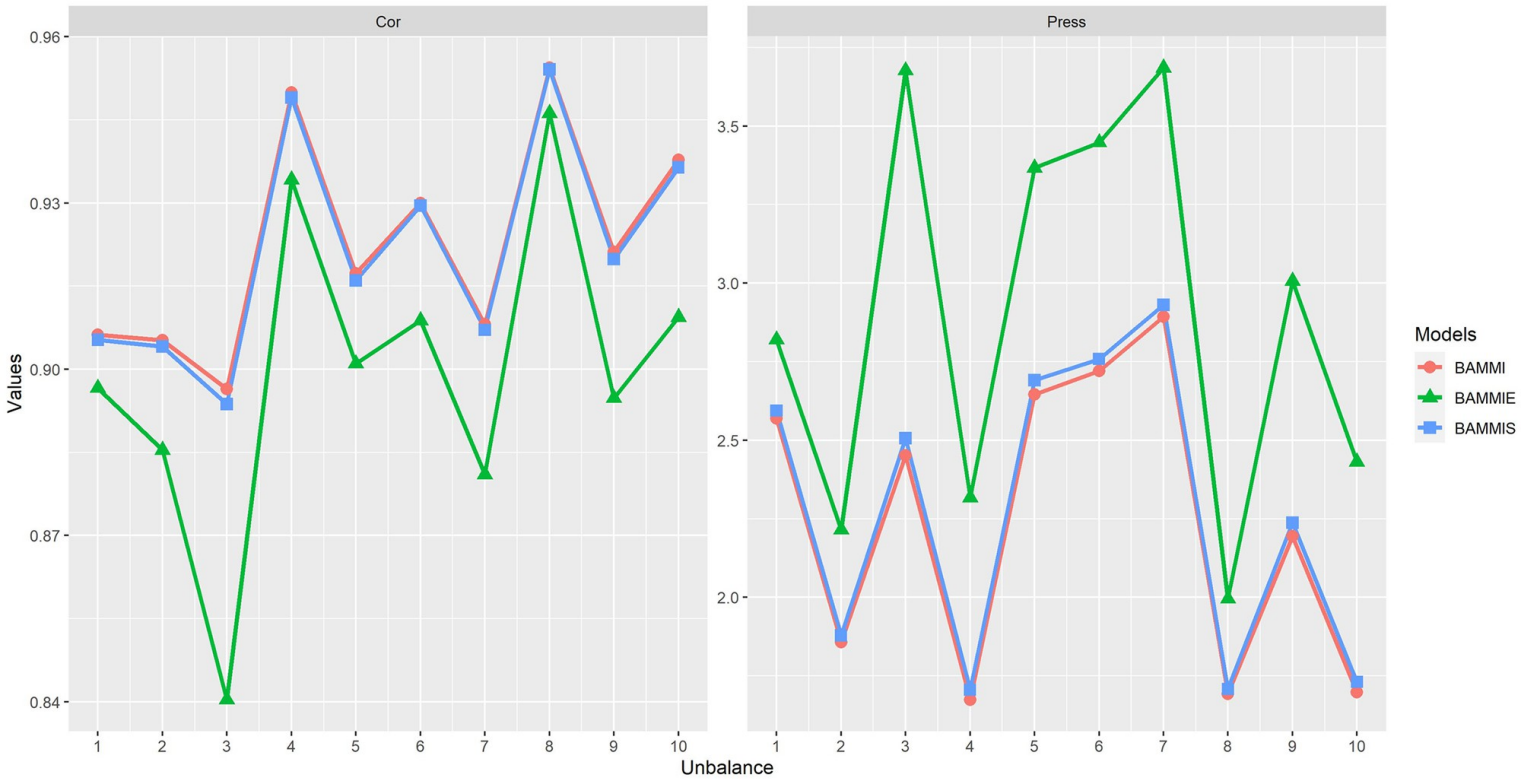
Ockham’s plot for the mean evaluation of the predictive capacity of the models based on the criteria of correlation between the observed and estimated values and the PRESS (RJMCMC algorithm).

The results of Cor and PRESS for the models fitted by the RJMCMC method are presented in Fig 9. For Cor, a mean behavior above 89% was observed, and individually (for the folds) the results were always above 84% for all models. For the PRESS criterion, the models that presented the best results were BAMMI-4, BAMMIS-4 and BAMMIE-8, and for Cor, the best result was presented by AMMI-8, regardless of the model. A similar pattern was observed between BAMMI and BAMMIS, both of which were better (in prediction) than BAMMIE, which also showed good performance.

### Real data

**Fig 10:**
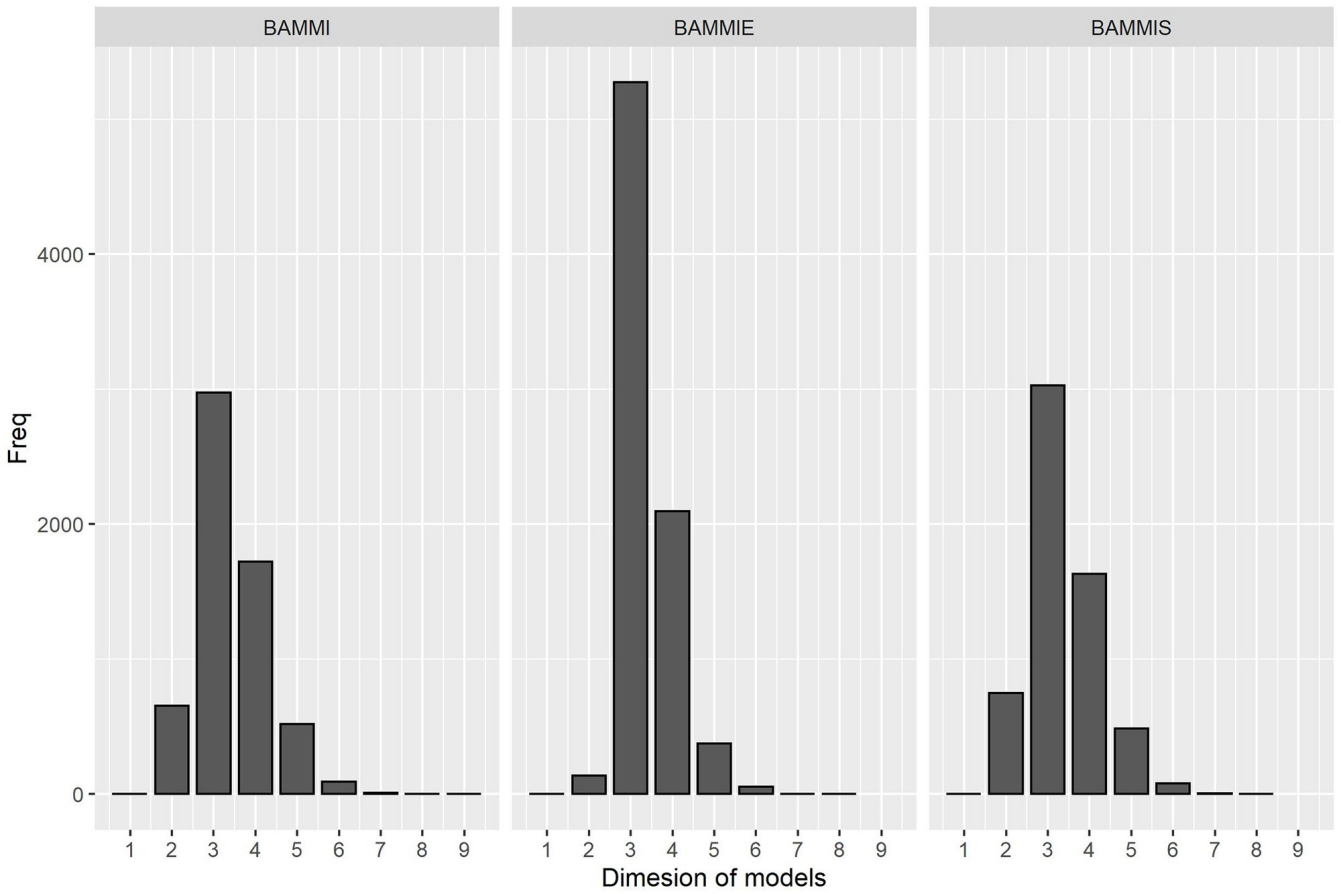
Frequency referring to the dimension (*t*) of the model accepted during the RJMCMC process (real data).

Analyses of actual data were performed using only the RJMCMC. The posterior estimates were obtained considering the marginal and conditional responses of the models, in a similar manner to what was done with the simulated data. In Fig 10, the histograms of the frequency of visits of the models in relation to the number of bilinear terms are presented.

Based on the frequencies of visits, AMMI-3 would be selected for the considered three versions of the AMMI model. Similar to in the study of simulated data, differences were observed in the frequency of the most likely model concerning the others.

Table 4 presents posterior means of singular values (for the conditional and marginal responses) and credibility regions estimated based on the marginal response. There were no accented differences between point estimates (conditional and marginal). Among the models, the mean estimates were close for the first three singular values, a fact that was also observed for the HPD interval at 95% of credibility.

**Table 4:** Point estimate and interval estimate (HPD at 95% probability) for singular values for the three Bayesian AMMI versions, using the RJMCMC algorithm.

	Parameter	Mean*	mean^#^	SD	LL	UL
BAMMI	*λ*_1_	5.3393	5.3370	0.3731	4.5805	6.0418
	*λ*_2_	4.1163	4.1121	0.4340	3.2611	4.9556
	*λ*_3_	3.2751	3.2712	0.4157	2.4368	4.0631
BAMMIE	*λ*_1_	5.2854	5.2855	0.3832	4.5404	6.0344
	*λ*_2_	3.9869	3.9857	0.4477	3.1284	4.8575
	*λ*_3_	3.1251	3.1251	0.4211	2.2802	3.9258
BAMMIS	*λ*_1_	5.3348	5.3363	0.3762	4.5747	6.0503
	*λ*_2_	4.0733	4.0758	0.4371	3.2221	4.9515
	*λ*_3_	3.2234	3.2234	0.4199	2.4129	4.0415

Mean*, marginal mean; mean^#^, conditional mean; SD, standard deviation; LL, lower limit; UL, upper limit.

Posterior means along with HPD regions for the residual and genotypic variance components, for the marginal and conditional responses, are presented in Table 5. The estimates, considering conditional and marginal responses, are practically the same within each model. Between the models, there were also no expressive differences in the estimates of the corresponding parameters.

**Table 5:** Point estimate and interval estimate (HPD at 95% probability) for the variance genotypic ($\sigma_{g}^{2})\;and\;variance\;residual\;(\sigma_{e}^{2}$) for the models, considering conditional and marginal responses, for the three Bayesian AMMI versions.

	Model	Par.	Mean	SD	LL	UL
BAMMI	Marginal	$\sigma_{g}^{2}$	0.3764	0.0981	0.198	0.5643
		$\sigma_{e}^{2}$	2.6761	0.0645	2.5528	2.8017
	Condicional	$\sigma_{g}^{2}$	0.3764	0.0989	0.2037	0.5643
		$\sigma_{e}^{2}$	2.6823	0.0585	2.5765	2.8016
BAMMIE	Marginal	$\sigma_{g}^{2}$	0.3811	0.0984	0.2126	0.5743
		$\sigma_{e}^{2}$	2.6785	0.0585	2.5644	2.7905
	Condicional	$\sigma_{g}^{2}$	0.3796	0.098	0.2081	0.5663
		$\sigma_{e}^{2}$	2.6817	0.0576	2.5749	2.7961
BAMMIS	Marginal	$\sigma_{g}^{2}$	0.3814	0.0996	0.2038	0.5789
		$\sigma_{e}^{2}$	2.6832	0.0581	2.5783	2.8003
	Condicional	$\sigma_{g}^{2}$	0.3814	0.0996	0.2038	0.5789
		$\sigma_{e}^{2}$	2.6832	0.0581	2.5783	2.8003

**Fig 11:**
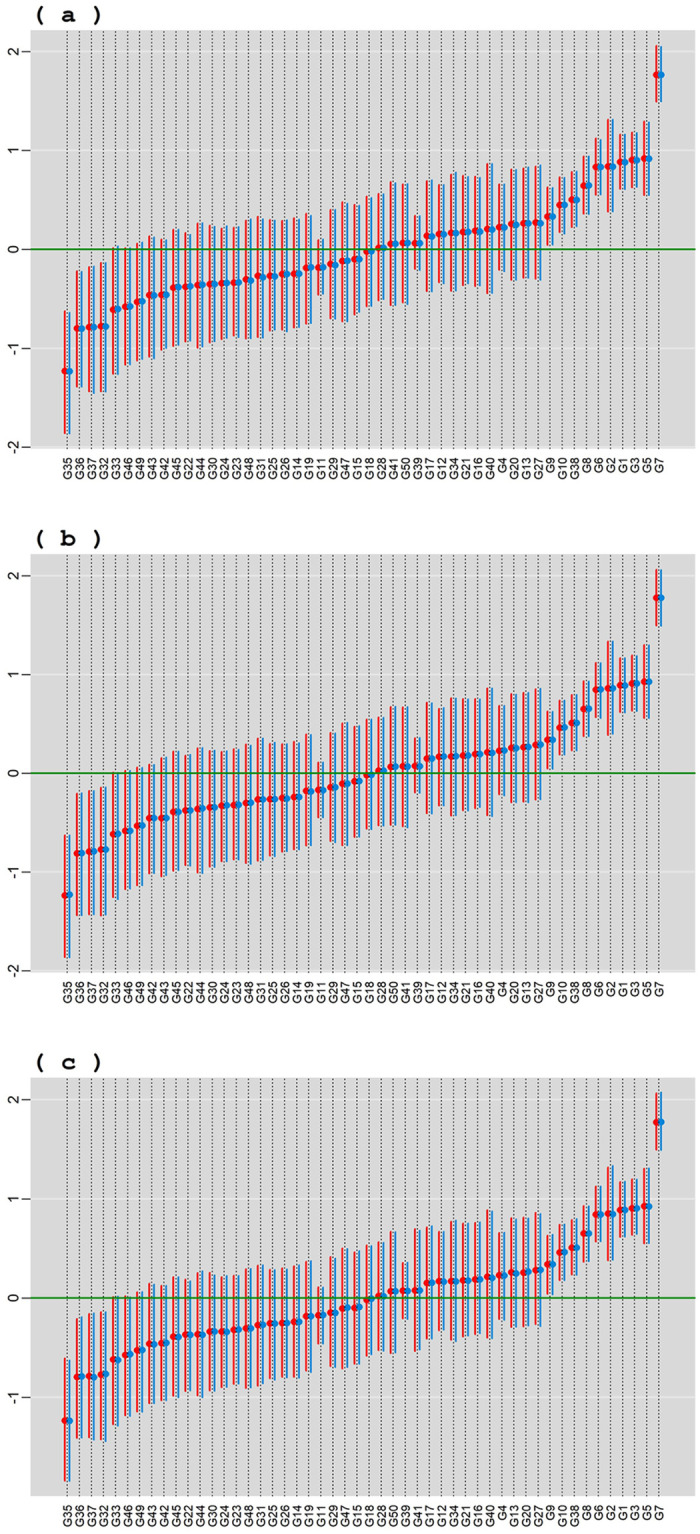
Posterior means and the 95% HPD intervals for the effect of genotypes, conditional response (blue color) and marginal responses (red color) referring to: a) BAMMI; b) BAMMIS; c) BAMMIE.

In Fig 11, posterior means and HPD intervals at 95% of credibility are presented, for the effect of genotypes, conditional response (blue color), and marginal response (red color). In both situations, the pattern in the classification of the effects was practically the same within each model. The inferences were also practically the same in both models with the same subgroup of genotypes (more productive) being selected {G9, G10, G38, G8, G6, G2, G1, G3, G5, G7}.

**Fig 12:**
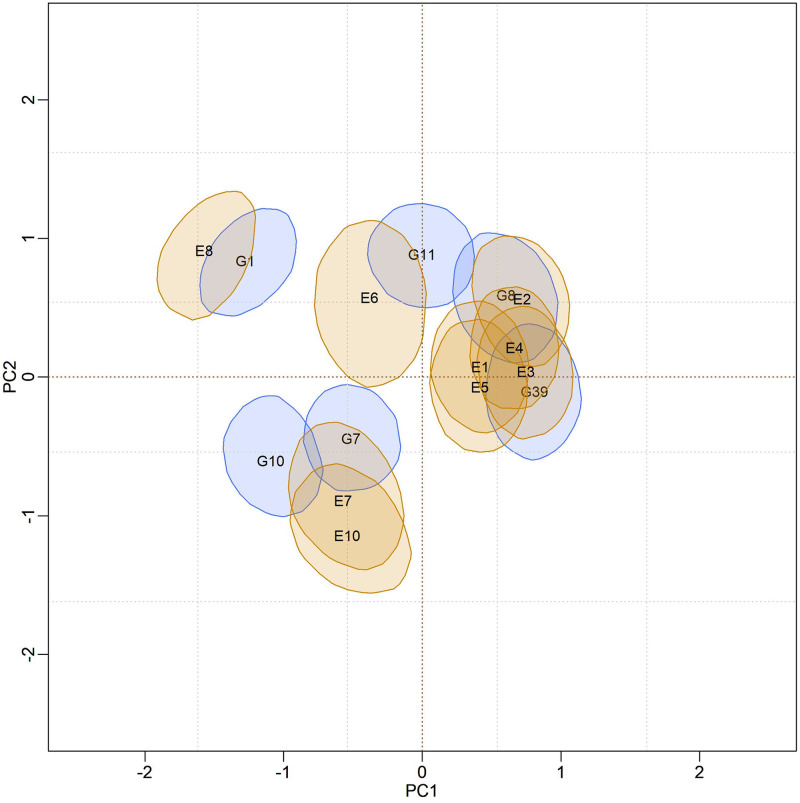
Bivariate credibility regions with 95% credibility for the genotypic and environmental scores of the BAMMI model, built based on the marginal response (AMMI3).

**Fig 13:**
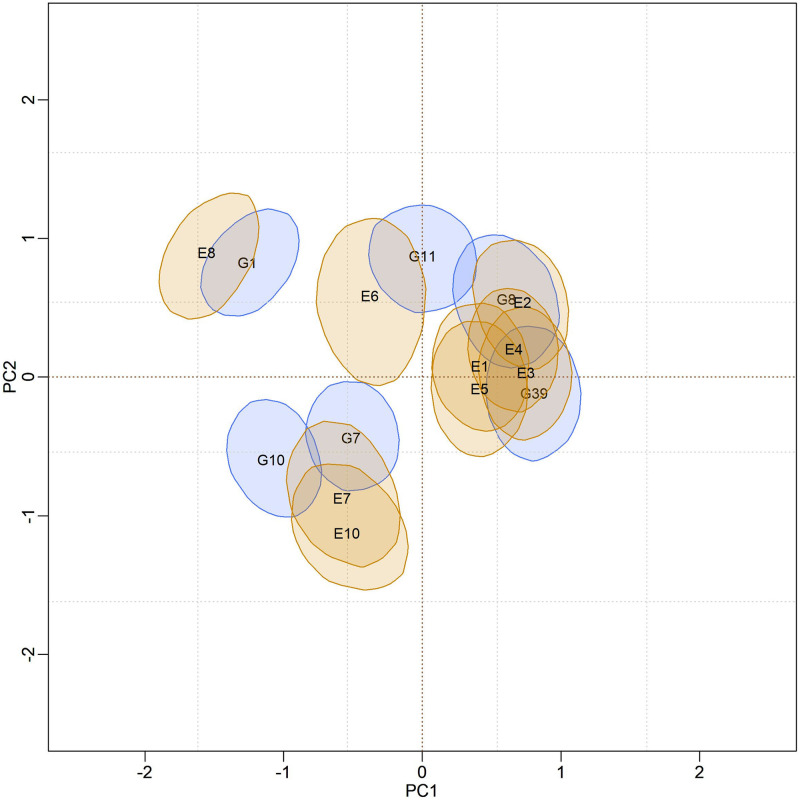
Bivariate credibility regions with 95% credibility for the genotypic and environmental scores of the BAMMIE model, built based on the marginal response (AMMI3).

**Fig 14:**
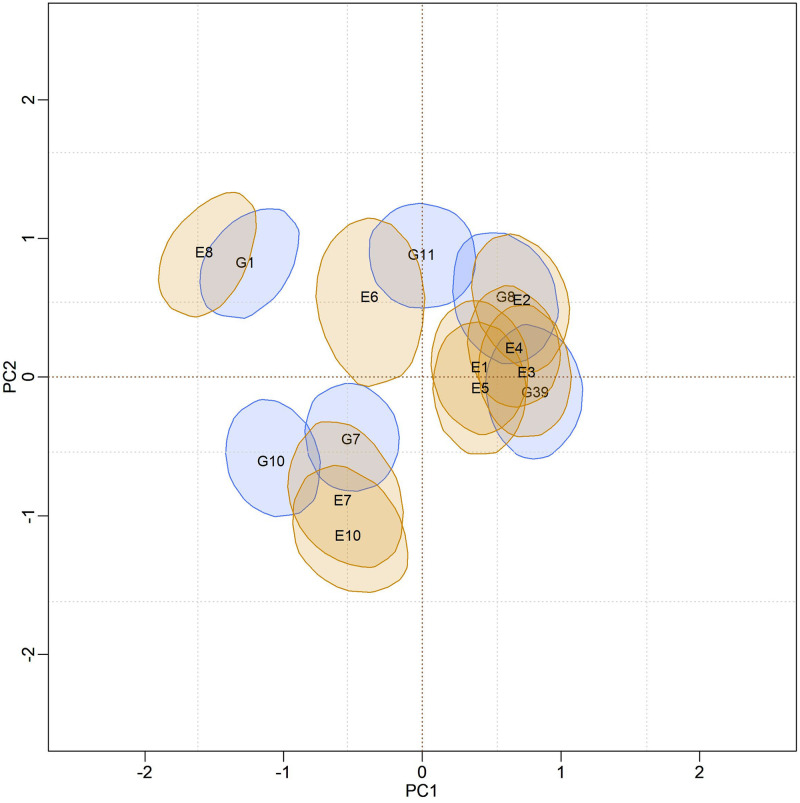
Bivariate credibility regions with 95% credibility for the genotypic and environmental scores of the BAMMIS model, built based on the marginal response (AMMI3).

Figs 12–14, are presented as biplots with 95% bivariate credibility regions for genotypic and environmental scores’ (to the AMMI3 model) marginal response. Here too, only the regions that do not contain the origin (0,0) were represented to simplify the interpretations; the genotypes and environments associated with these regions have an important contribution to the GEI effect. By visually inspecting positions and overlaps between credibility regions, the same homogeneous genotype subgroups, {G1}, {G7, 10}, {G11} and {G8, G39}, can be suggested for all models. The remaining genotypes (not represented) form another subgroup, and they are interpreted as stable. The same interpretation can be made in relation to the environments, so that five subgroups can be identified, namely: {E8, E6}, {E7, E10}, {E1, E2, E3, E4, E5} and {E9}. The region associated with the E9 environment encompasses the origin; thus, it is considered stable, and the other environments significantly contribute to the GEI effect.

The adaptability of genotypes (or subgroup of genotypes) to environments (or subgroups of environments) can also be suggested from the biplot visualization. This analysis can be performed by observing positions and overlaps between regions of credibility for genotypic and environmental scores in the quadrants referring to the PC1 and PC2 axes. It is noteworthy that these inferences would be almost identical in both biplots.

## Points considered for exemplifying the method

In the introduction, we pose two fundamental questions concerning the work of plant breeders. We previously described some differences between the three versions of the AMMI model fitted with MCMC and RJMCMC. We will now highlight differences (or similarities) between the two adjustment methods (MCMC × RJMCMC) for simulated and real data in the analysis of adaptability and phenotypic stability.

### Stability analysis for simulated data

Estimates for the singular values of selected models (from information criteria) and the most likely models using RJMCMC were not very outliers. However, as already reported, it was observed that in the assessment of the predictive capacity, the models adjusted by the RJMCMC method performed better than those adjusted by the MCMC method.

The selection and recommendation of genotypes are one of the main objectives of plant breeding programs. The identification of stable genotypes makes it possible to minimize the interaction effect if they have interesting yields. We can compare the main effects of genotypes between models fitted with MCMC and RJMCMC by looking at Fig 3 and the figures that are in the S1–S3 Figs. From the graphical analysis, we found that the inferences do not change even between the different AMMI versions. The best genotypes in terms of the main effect are the same {G1, G12, G4, G10, G13, G11}.

The second step in this evaluation would be to observe which of these genotypes would be stable by comparing the versions of the models with MCMC and RJMCMC. Considering MCMC models (S4–S6 Figs), G4 is stable in all the model AMMI versions, while G13 is stable also for BAMMIS and G12 for BAMMIE. Therefore, among the best genotypes (considering main effects), these would have a broad recommendation, respectively, for BAMMI, BAMMIS, and BAMMIE.

When analyzing the biplots of the models for the RJMCMC analyses, it is noticed that G4 is also stable in all of them. The only difference is that G13 is also considered stable for BAMMI-E, which differs from the analysis using MCMC. But, in general, the inferences between these two approaches converge.

### Adaptability to simulated data

Another way to mitigate the effect of the GEI is to take advantage of its positive effect. For this, it is necessary to evaluate the biplot and interpret positions and overlaps of genotypic and environmental credibility regions.

In the biplots of models fitted with MCMC (S4–S6 Figs), the visual analysis suggests separable subgroups concerning the interaction effect. For the three versions of the model, the GEI standard, in general, remains in the biplot representation. It is observed that for models fitted with RJMCMC the patterns are similar (Figs 5–7). The similarity is also observed when comparing the biplots of equivalent models (MCMC × RJMCMC). This shows us that the analysis of adaptability and stability when considering the different methods, in general, offers the same inferences. Concerning prediction, a slight advantage was seen for the RJMCMC method. In addition, the computational analysis time for the adjustment proved to be much more feasible.

### Stability analysis for real data

We exemplified the RJMCMC method with real data and observed, concerning the main effect, that the subgroup {G9, G10, G38, G8, G6, G2, G1, G3, G5, G7} stood out, and even though this inference is independent of the model (BAMMI, BAMIS or BAMMIE). In addition, we saw that G7 was the one that presented the highest value for the main effect since its HPD region does not overlap with any other (Fig 11).

In the biplot analysis (Figs 12, 13, and 14), we also observed that the genotypes G1, G7, 10, G11, G8, and G39 have significant contributions to the GEI the others and were considered stable since their bivariate credibility regions include the origin (0,0). This inference was common to both versions (BAMMI, BAMMIS, and BAMMIE). Thus, among the best genotypes for the main effect, the subgroup {G2, G3, G5, G6, G9, G38}, formed by stable genotypes, has a broad recommendation for all environments tested.

**Fig 15:**
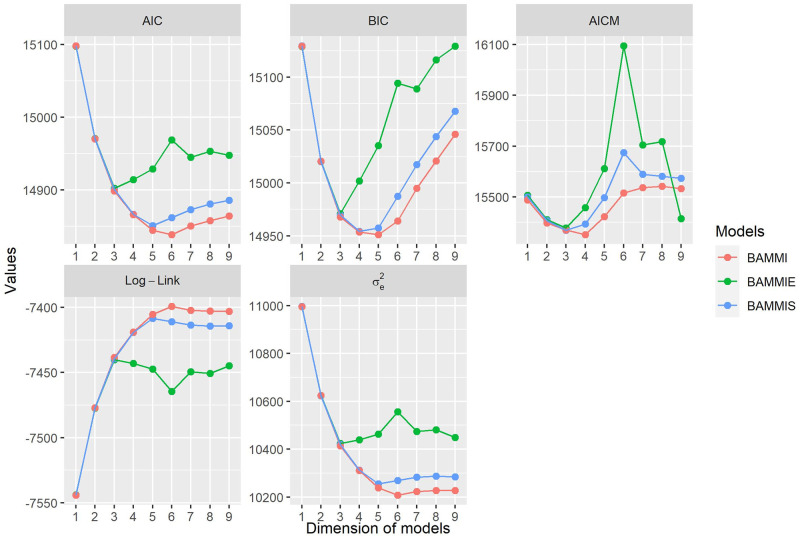
Ockham’s plot for the BAMMI, BAMMIS, and BAMMIE models for the AIC, AICM, and BIC information criteria for real data. The behaviors of log-likelihood (log-like) and residual variance (*σ*_*e*_^2^) are also presented.

For comparison purposes, fitted the models by the MCMC method and used information criteria for model selection. Fig 15 presents the results of model selections obtained by AIC, BIC, and AICM. As additional criteria, the behaviors of log-likelihood and residual variance are presented. In this sense, models with lower residual variance are preferable, while higher log-likelihood indicates the best model.

The AICM criterion indicated that the BAMMIS and BAMMIE models with three bilinear terms (BAMMIS-3 and BAMMIE-3) would be the best, while for the BAMMI four principal components (BAMMI-4) would be necessary to obtain the optimal model. Using BIC and AIC criteria, we would select different models for each AMMI version. For the AIC criterion, we would have BAMMIE-3, BAMMIS-5, and BAMMI-6; while the BIC criterion pointed out the models: BAMMIE-3, BAMMIS-4, and BAMMI-5.

**Fig 16:**
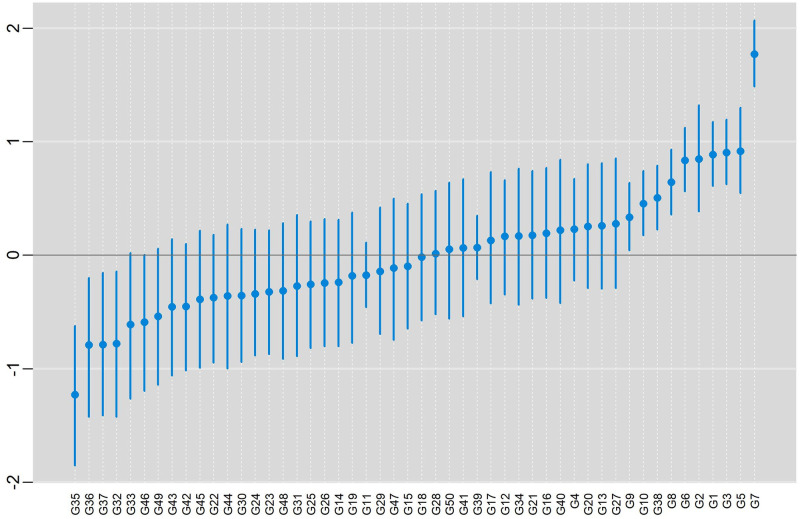
Posterior means and the 95% HPD intervals for the effect of genotypes, considering the BAMMIE-3 model, for real data using the MCMC method.

**Fig 17:**
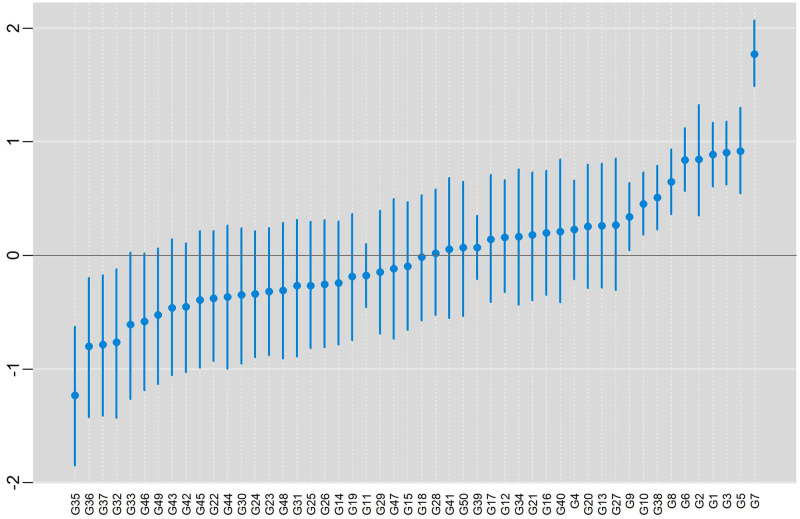
Posterior means and the 95% HPD intervals for the effect of genotypes, considering the BAMMIS-3 model, for real data using the MCMC method.

**Fig 18:**
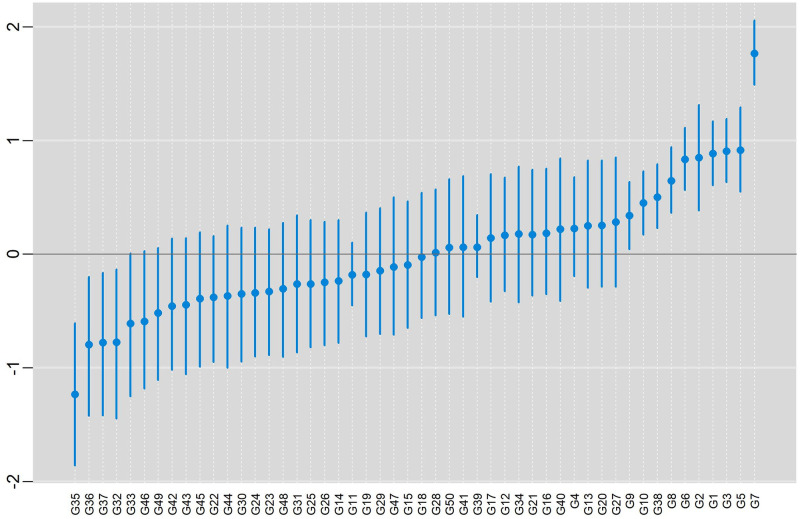
Posterior means and the 95% HPD intervals for the effect of genotypes, considering the BAMMI-4 model, for real data using the MCMC method.

In Figs 16–18 present posterior means and HPD regions, at 95% credibility, for genotype main effects of the models BAMMIE-3, BAMMIS-3, and BAMMI-4, respectively. To make these inferences, we considered the models selected by the AICM criterion, remembering that for the BAMMIE, the model selected by the criteria used was BAMMIE-3.

As you can see, the inferences regarding the main effect (ranking and credibility regions) do not change from one version to another. When we compare these results with those obtained with the models adjusted with RJMCMC (Fig 11), the inferences are the same, with the subgroup {G9, G10, G38, G8, G6, G2, G1, G3, G5, G7} highlighting and G7 being the best genotype. This behavior was also observed for models adjusted using AIC or BIC (S4 Appendix).

**Fig 19:**
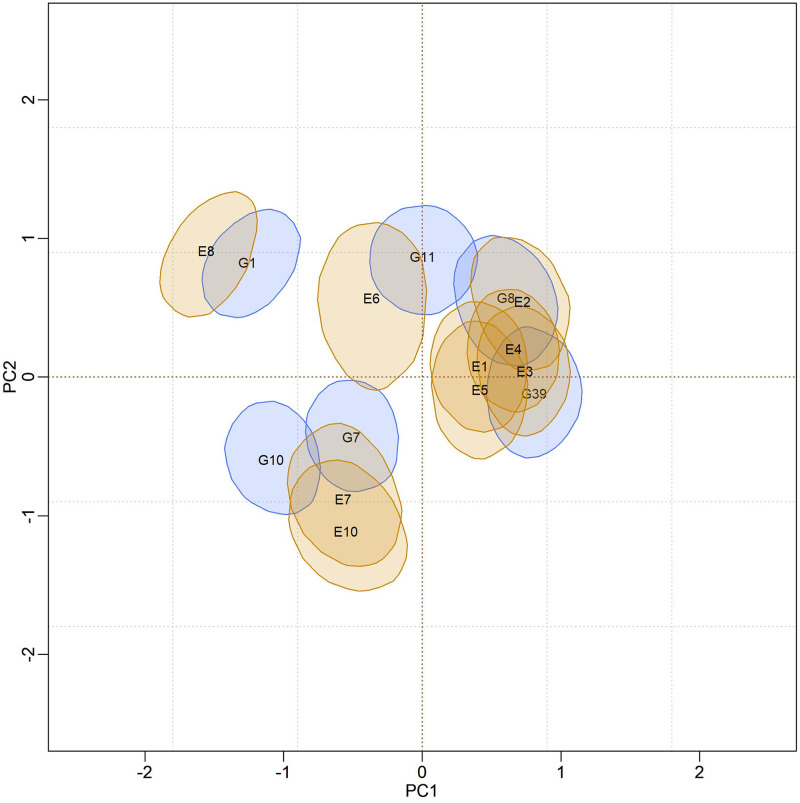
Bivariate credibility regions at 95% probability for the genotypic and environmental scores of the BAMMIE-3 model for real data, adjusted using the MCMC method. Only regions whose regions do not contain the origin (0.0) were plotted.

**Fig 20:**
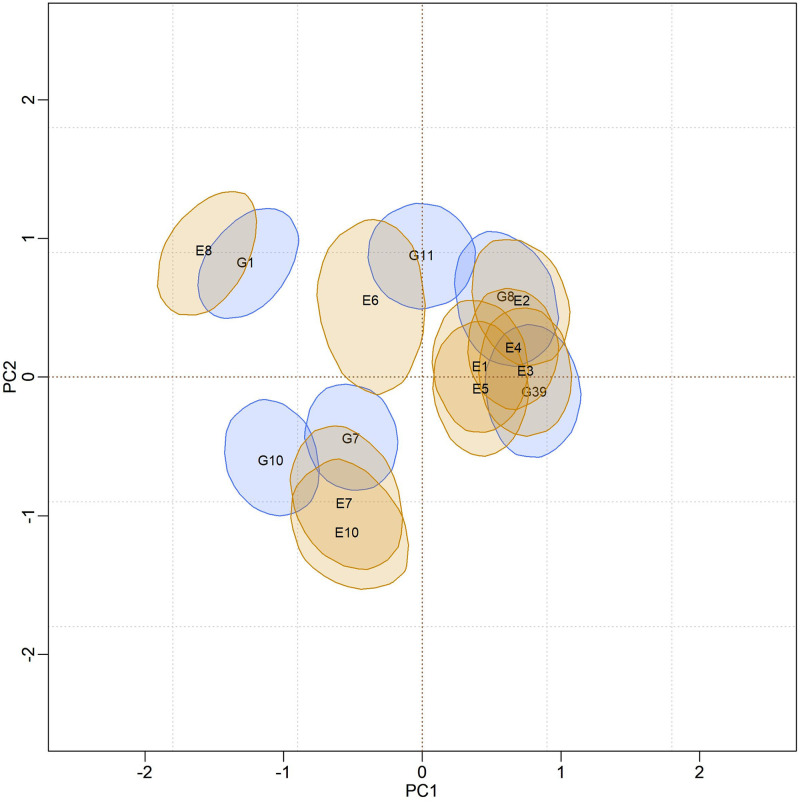
Bivariate credibility regions at 95% probability for the genotypic and environmental scores of the BAMMIS-3 model for real data, adjusted using the MCMC method. Only regions whose regions do not contain the origin (0.0) were plotted.

**Fig 21:**
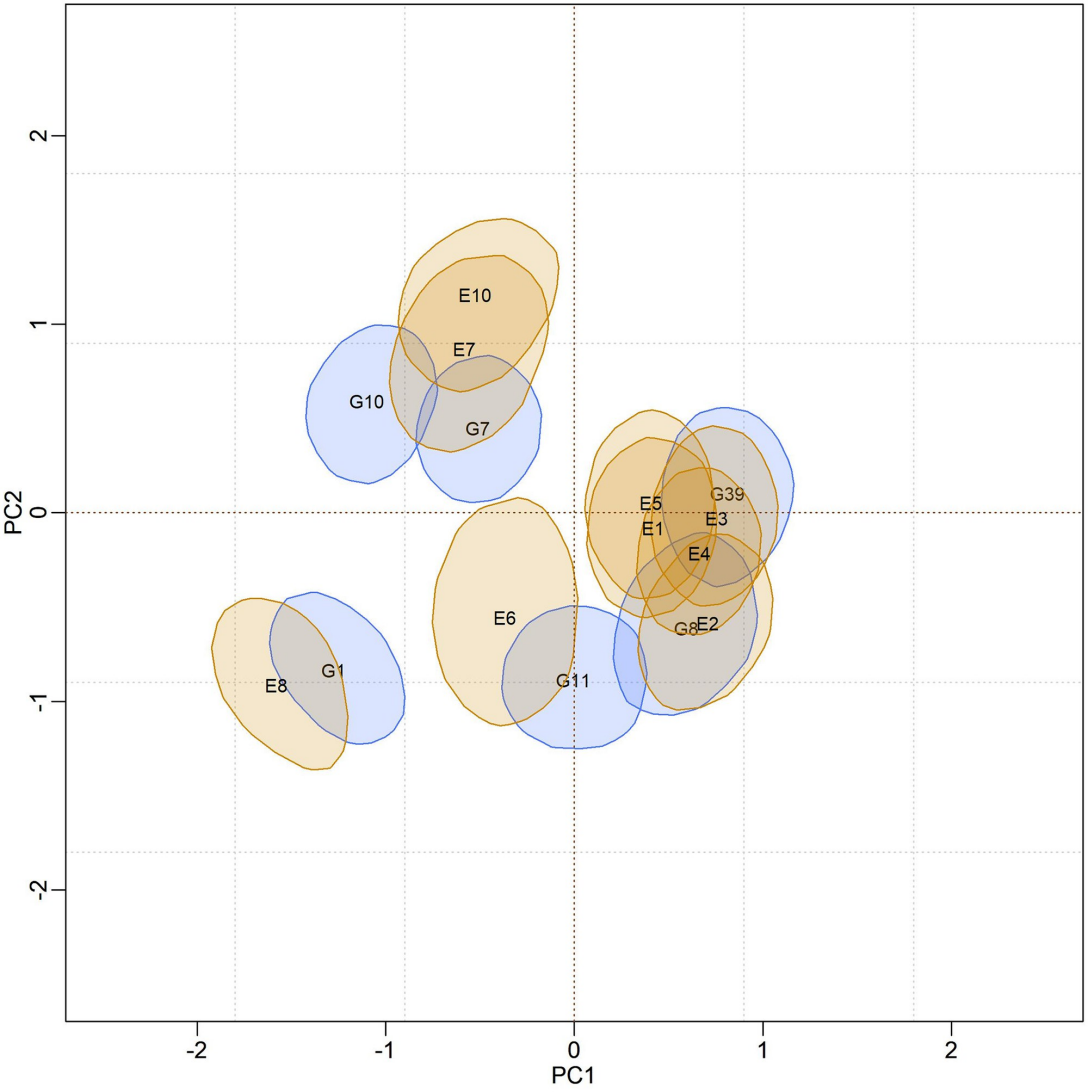
Bivariate credibility regions at 95% probability for the genotypic and environmental scores of the BAMMI-4 model for real data, adjusted using the MCMC method. Only regions whose regions do not contain the origin (0.0) were plotted.

The biplots for the models selected from the AICM method are shown in Figs 19–21. We observe that the positions and overlaps of the credibility regions in the biplots of the three models are practically identical. Furthermore, there are no significant discrepancies when comparing these biplots with those of Figs 12–14, built from the fit using the RJMCMC method. So, the inferences are also the same.

### Adaptability in real data

For models fitted with the RJMCMC method, we highlighted the following separable homogeneous subgroups of unstable genotypes {G1}, {G7, 10}, {G11} and {G8, G39}. Genotypes in each subgroup are similar concerning the effect of GEI. We point out that the same subgroups can be identified regardless of the model (BAMMI, BAMMIS, and BAMMIE). Therefore, considering the RJMCMC biplot analysis (Figs 12–14), it is possible to make the following recommendations: G1 genotype for the E8 environment, the subgroup of genotypes {G8, G39} for the subgroup of environments {E1, E2, E3, E4, E5} and subgroup {G7, G10} to environments E7 and E10.

This same result can be inferred from the biplot analysis for the model adjusted by the MCMC method. As already highlighted, the advantages of using the RJMCMC are related to the probabilistic nature of this proposal, such as its feasibility, since the use of information criteria in the selection of models is exhaustive and may become unfeasible. Concerning the analysis of stability and adaptability, we did not find significant differences when considering the different AMMI versions, depending on the a priori distribution adopted for the singular values. Our objective, in this sense, was to show that the RJMCMC can be applied to any Bayesian version of the AMMI model present in the literature.

## Discussion

In analyzes using linear-bilinear models, it is important to determine how many bilinear terms should be retained to describe the pattern of interaction. In the frequentist method, this is usually done through parametric tests based on the F distribution or using cross-validation schemes [5, 17, 19]. The question of the dimensionality of the model naturally arises from the Bayesian perspective, although this subject is still not very widespread in the Bayesian analysis of linear-bilinear models and, in particular, in the Bayesian-AMMI. Most approaches have not focused on the selection of models, but the assessment of stability and adaptability, using credibility regions incorporated in the biplot [11, 13, 42, 43].

The most recent works on model selection in the Bayesian analysis of AMMI and GGE were carried out by Silva et al. [24] and Oliveira et al. [27], respectively. The analyzes presented by these authors can be considered Bayesian versions of the frequentist proposal of Cornelius and Crossa [25]. One of the reasons for using shrinkage estimators is the possibility of dispensing with the use of any criteria to determine the number of bilinear parameters to be retained in the analysis. The use of shrinkage estimators provides better adjustments in terms of prediction. But the method does not necessarily lead to a more parsimonious choice in terms of the model dimension. In this sense, we argue in favor of the need to think about procedures for selecting models, even when specific priors are used for the components of singular values, as is the case of the BAMMIS and BAMMIE models that were discussed in this study. Liu [10] offers a rich discussion on the selection of models for the Bayesian-AMMI, using information criteria BIC and AIC, and Bayes factor to determine the best model.

Despite their popularity, information criteria such as AIC and BIC require an exhaustive evaluation of all possible models. This fact can make applications unfeasible when the model space is large and, therefore, only in limited circumstances are indicated. Furthermore, the Bayes factor, as Liu [10] argues, is sensitive to the choice of priors, and choosing an ideal prior is a difficult task (although this criterion has not been used in this study). It is also noted that different criteria, in general, tend to choose different models (Fig 1). In this sense, it seems more reasonable to opt for the AICM criterion, which, for the conditional inference, proved to be more consistent, for selecting models with the same dimension. In turn, the use of the RJMCMC algorithm, allows both the adjustment and determination of the model dimension simultaneously. It is one of the most popular methods involving transdimensional Markov chains and has been applied in several contexts [29, 34, 44–46].

In assessing the predictive ability, we consistently observed good properties for the three versions of the AMMI model. As noted, the results of the models fitted by the two methods do not coincide. The results obtained with the RJMCMC were always superior to the Gibbs approach both by the PRESS criterion and by the Correlation. This was also observed for the conditional response of each model (Figs 8 and 9 and S6 Table).

Another point to be highlighted was the use of different priors related to the parameter $\sigma_{\lambda_{k}}^{2}$. In the BAMMI model, as in Crossa et al. [11] and Oliveira et al. [13], a prior can be considered as an approximation of the principle of insufficient reason. The BAMMIS proposed by Silva et al. [24] was obtained by assigning the Jeffreys prior [47]. This prior is based on the principle of invariance. Other suppositions were investigated by Liu [10], where he showed that different suppositions could lead to different results, as verified in this study as well. Despite the wide use of non-informative priors, they may not represent the current state of available information about the problem. In most cases, they are used only as reference distributions, that is, as a default option in the absence of any available information. Thus, among non-informative prior calls, there are some that are more useful and, therefore, frequently used. But depending on the situation, it would be incorrect to say that these are less informative than others. In this sense, the use of a prior based on the principle of maximum entropy [27] provided us with a theoretical justification for performing inference with less informative priors. Using maximum entropy priors can also be useful in situations of incomplete or doubtful information [48–51].

With simulated data, we observed that, in general, the choice of priors did not affect the pattern of the marginal or conditional response of the model in the biplot. The prior based on the principle of maximum entropy (BAMMIE) provided a more parsimonious choice, but, with few exceptions, it did not change the patterns of the marginal bivariate credibility regions obtained with other priors. This choice is convenient in the example, as the breeder tends to prefer biplot representations to evaluate genotype responses in different environments. The biplot analysis facilitates the work of selection and recommendation of superior cultivars. Although no expressive discrepancies were found, opting for the marginal biplot could be interesting as it produces less Monte Carlo error. However, the conditional biplot determines a greater degree of freedom for the error.

From S8 and S9 Tables, it is possible to infer that the models, in general, performed well in correctly identifying stable and unstable genotypes (based on the simulated scenario). This behavior was verified for the fit using only the Gibbs sampler as well as the RJMCMC method. For real data, the most likely model was the one with three retained multiplicative terms (AMMI3) for all AMMI versions (BAMMI, BAMMIS, and BAMMIE). This became more evident for BAMMIE, as indicated by noting the marked difference between the frequency of “visits” (Fig 10). Furthermore, the biplot patterns for the three models are practically indistinguishable. Therefore, in theory, for a graphical evaluation of patterns, BAMMIE could be preferable. The justification is based on the singular value decomposition property that the first axes capture more information. BAMMIE better estimates the first two singular values, reducing the estimates of those related to higher dimensions to zero. Furthermore, the most popular biplot analysis is the AMMI-2 biplot analysis, which is systematically used even when the selected model dimension is greater than two. In addition, the use of maximum entropy priors makes the analysis more flexible, justifying the shrinkage effect without the need of imposing restrictions on the prior to avoid obtaining inappropriate marginal posterior distributions, as presented in Silva et al. [24]. This aspect was also emphasized by Oliveira et al. [27] who reported that the use of the principle of maximum entropy brings greater stability to the algorithm and simplifies the estimation of parameters.

The issues raised here present a good reason to choose the RJMCMC, although the dataset used in our approach is small. In this sense, the computational time of analysis with RJMCMC is much more attractive. As can be seen from S7 Table, the selection of models by information criteria involves procedures whose total computational time (in minutes) is at least four times longer compared to the RJMCMC method. Another justification for using RJMCMC, as mentioned in this text, would be that this method leads to a natural extension of the Markov chain theory.

Other RJMCMC approaches could be used. We chose the one that seemed the simplest to us and didn’t bother comparing different methods trying to find the best one. Our objective was to implement RJMCMC directly in the model fit and to show that the method is a more viable alternative than the use of information criteria. However, the comparison of different RJMCMC methods, aiming to find the best one, is very interesting and can lead to greater efficiency in the adjustment. Another option would be to use non-MCMC methods, which could solve problems such as poor mixing and the difficulty of diagnosing convergence. In some situations, there is evidence that non-MCMC methods can be more efficient than MCMC algorithms [52, 53]. Fearnhead [54] emphasizes this idea, reviewing three alternatives to MCMC methods: Importance Sampling, Forward-Backward algorithm, and Sequential Monte Carlo (SMC), discussing the efficiency and limitations of these procedures. The use of non-MCMC procedures could be an option to make the Bayesian analysis of multiplicative models more flexible. For this, further studies are needed and will be the subject of future research.

## Supporting information

S1 Appendix The complete posterior conditional distributions for the models. (PDF)

S2 Appendix Maximum entropy prior. (PDF)

S3 Appendix Real data set. (CSV)

S4 Appendix Posterior summaries for AMMI models with the MCMC method selected by AIC and BIC. (PDF)

S1 Table Singular values were obtained through the SVD of the GEI simulated. (PDF)

S2 Table Estimates for the variance components of the genotypic effect and residual variance component, referring to the AMMI model (Gibbs algorithm). (PDF)

S3 Table Posterior means and HPD intervals (at 95%) for the genotypic effects of the model BAMMI (conditional and marginal), RJMCMC algorithm. (PDF)

S4 Table Posterior means and HPD intervals (at 95%) for the genotypic effects of the model BAMMIS (conditional and marginal), RJMCMC algorithm. (PDF)

S5 Table Posterior means and HPD intervals (at 95%) for the genotypic effects of the model BAMMIE (conditional and marginal), RJMCMC algorithm. (PDF)

S6 Table Correlation and PRESS for the models (RJMCMC). (PDF)

S7 Table Total computational time, of analysis of the models using Gibbs and RJMCMC algorithm. (PDF)

S8 Table Classification of the pattern of genotypic scores by biplot AMMI2 representation based on the simulated scenario (Gibbs algorithm). (PDF)

S9 Table Classification of the pattern of genotypic scores by biplot AMMI2 representation based on the simulated scenario (RJMCMC algorithm). (PDF)

S1 Fig Genotypic effect of the BAMMI model (Gibbs algorithm). (PDF)

S2 Fig Genotypic effect of the BAMMIS model (Gibbs algorithm). (PDF)

S3 Fig Genotypic effect of the BAMMIE model (Gibbs algorithm). (PDF)

S4 Fig Bivariate credibility regions for the genotypic and environmental scores of the BAMMI model (Gibbs algorithm). (PDF)

S5 Fig Bivariate credibility regions for the genotypic and environmental scores of the BAMMIE model (Gibbs algorithm). (PDF)

S6 Fig Bivariate credibility regions for the genotypic and environmental scores of the BAMMIS model (Gibbs lgorithm). (PDF)
