# Supplementary material for: Use of the reversible jump Markov chain Monte Carlo algorithm to select multiplicative terms in the AMMI-Bayesian model
Source: PLoS One. 2023 Jan 3;18(1):e0279537. doi: 10.1371/journal.pone.0279537 (PMC9810207; doi:10.1371/journal.pone.0279537)
Supplement: S1 Appendix — (PDF) [file pone.0279537.s001.pdf]

S2 Appendix

Maximum entropy priority for $\sigma^2_{\lambda_k}$

Our objective here is to construct a prior based on the maximum entropy principle Jaynes [1] for the variance component $\sigma^2_{\lambda_k}$.

According to information theory, the distribution that best represents the current state of information is obtained by maximizing the Shannon entropy [2]. For the specification of the prior, considering the Shannon entropy function, we assume that $E(\sigma^2_k) \geq 0$, this being the only restriction we use.

For algebraic convenience, we choose to work with the precision $\tau_{\lambda_k} = \frac{1}{\sigma^2_{\lambda_k}}$, noting that: $E(\sigma^2_{\lambda_k}) \geq 0 \rightarrow E(\tau_{\lambda_k}) \geq 0$.

Using the Lagrangian function have the function that maximizes the entropy is:

$$\int_0^{+\infty} \tau_{\lambda_k} p(\tau_{\lambda_k}) d\tau_{\lambda_k} = \vartheta \ \rightarrow \Lambda \left[\int_0^{+\infty} \tau_{\lambda_k} p(\tau_{\lambda_k}) - \vartheta \right].$$

The general expression of the maximum entropy distribution generated from the considered bond is [3]:

$$P(\tau_{\lambda_k}) = \frac{e^{-\Lambda\tau_{\lambda_k}}}{\int_0^{+\infty} e^{-\Lambda\tau_{\lambda_k}} d\tau_{\lambda_k}}. \tag{1}$$

where:

$$\int_0^{+\infty} e^{-\Lambda\tau_{\lambda_k}} d\tau_{\lambda_k} = \int_0^{+\infty} \frac{\Lambda}{\Lambda} e^{-\Lambda\tau_{\lambda_k}} d\tau_{\lambda_k} = \frac{1}{\Lambda} \int_0^{+\infty} \Lambda e^{-\Lambda\tau_{\lambda_k}} d\tau_{\lambda_k} = \frac{1}{\Lambda}; \Lambda > 0.$$

Thus:

$$P(\tau_{\lambda_k}) = \frac{e^{-\Lambda\tau_{\lambda_k}}}{1/\Lambda} = \Lambda e^{-\Lambda\tau_{\lambda_k}}.$$

Thus we have the function of the density of the variable τ_{λ_k}. However, the

interest and in the density of $\sigma^2_{\lambda_k}$ and $\sigma^2_{\lambda_k} = 1/\tau_{\lambda_k}$. The density of $\sigma^2_{\lambda_k}$ is obtained by the Jacobian of the transformation.

$$f_{\sigma^2_{\lambda_k}}\left(\sigma^2_{\lambda_k}\right) = \left|\frac{d}{d\sigma^2_{\lambda_k}} g^{-1}\left(\sigma^2_{\lambda_k}\right)\right| f_{\tau_{\lambda_k}}\left(g^{-1}\left(\sigma^2_{\lambda_k}\right)\right) I_D\left(\sigma^2_{\lambda_k}\right)$$

with: $\sigma^2_{\lambda_k} = g\left(\tau_{\lambda_k}\right) = 1/\tau_{\lambda_k} \Rightarrow \left(\sigma^2_{\lambda_k}\right)^{-1} = \tau_{\lambda_k}$, then

$$\frac{d}{d\sigma^2_{\lambda_k}} g^{-1}\left(\sigma^2_{\lambda_k}\right) = \frac{d}{d\sigma^2_{\lambda_k}}\left(\frac{1}{\sigma^2_{\lambda_k}}\right) = -1/\left(\sigma^2_{\lambda_k}\right)^2.$$

Soon:

$$f\left(\sigma^2_{\lambda_k}\right) = \Lambda e^{-\Lambda/\sigma^2_{\lambda_k}} \left|-1/\left(\sigma^2_{\lambda_k}\right)^2\right| = \frac{\Lambda}{\Gamma(1)}\left(\sigma^2_{\lambda_k}\right)^{-(1+1)} e^{e^{-\Lambda/\sigma^2_{\lambda_k}}};$$

where: $\Gamma(\cdot)$ is the gamma function.

Thus $\sigma^2_{\lambda_k} \sim Inv - gamma(a, b)$ where $a = 1$, $b = \Lambda$ e $\Lambda > 0$.

References

1. Jaynes ET. Information Theory and Statistical Mechanics. Phys Rev. 1957;106(4):620–30. Available from: https://link.aps.org/doi/10.1103/PhysRev.106.620

2. Shannon CE. A mathematical theory of communication. Bell Syst Tech J. 1948;27(3):379–423.

3. Cover TM, Thomas JA. Elements of Information Theory. Elements of Information Theory. 2006. 774 p.
