# Supplementary material for: Use of the reversible jump Markov chain Monte Carlo algorithm to select multiplicative terms in the AMMI-Bayesian model
Source: PLoS One. 2023 Jan 3;18(1):e0279537. doi: 10.1371/journal.pone.0279537 (PMC9810207; doi:10.1371/journal.pone.0279537)
Supplement: S2 Appendix — (PDF) [file pone.0279537.s002.pdf]

S1 Appendix

The complete posterior conditional distributions for the models.

a) Complete conditional a posteriori distribution for $\boldsymbol{\beta}$:

$$P(\boldsymbol{\beta} \mid \ldots) \propto |\mathbf{I}\sigma_e^2|^{-\frac{1}{2}} exp\left\{-\frac{1}{2\sigma_e^2}(\mathbf{y} - \theta)^\top(\mathbf{y} - \theta)\right\} P(\boldsymbol{\beta})$$

$$P(\boldsymbol{\beta} \mid \ldots) \propto exp\left\{-\frac{1}{2\sigma_e^2}(\mathbf{A}_1 - \mathbf{X}_1\boldsymbol{\beta})^\top(\mathbf{A}_1 - \mathbf{X}_1\boldsymbol{\beta})\right\} \quad (1)$$

Developing the quadratic form of the numerator of the expression (1) between the braces, we obtain:

$$(\mathbf{A}_1 - \mathbf{X}_1\boldsymbol{\beta})^\top(\mathbf{A}_1 - \mathbf{X}_1\boldsymbol{\beta}) = \mathbf{A}_1^\top\mathbf{A}_1 - \mathbf{A}_1^\top\mathbf{X}_1\boldsymbol{\beta} - \boldsymbol{\beta}^\top\mathbf{X}_1^\top\mathbf{A}_1 + \boldsymbol{\beta}^\top\mathbf{X}_1^\top\mathbf{X}_1\boldsymbol{\beta}.$$

From algebraic calculations and, still, observing that terms not dependent on $\boldsymbol{\beta}$ can be absorbed by the normalization constant, we arrive at the expression:

$$P(\boldsymbol{\beta} \mid \ldots) \propto exp\left\{-\frac{1}{2\sigma_e^2}(-2\boldsymbol{\beta}^\top\mathbf{X}_1^\top\mathbf{A}_1 + \boldsymbol{\beta}^\top\mathbf{X}_1^\top\mathbf{X}_1\boldsymbol{\beta})\right\}$$

In addition, completing squares concerning a multivariate normal distribution, we have:

$$P(\boldsymbol{\beta} \mid \ldots) \propto exp\left\{-\frac{1}{2\sigma_e^2}(\boldsymbol{\beta} - (\mathbf{X}_1^\top\mathbf{X}_1)^{-1}\mathbf{X}_1^\top\mathbf{A}_1)^\top(\mathbf{X}_1^\top\mathbf{X}_1) \times \right.$$
$$\left. \times (\boldsymbol{\beta} - (\mathbf{X}_1^\top\mathbf{X}_1)^{-1}\mathbf{X}_1^\top\mathbf{A}_1)\right\}.$$

Thus, the complete posterior distribution for $\boldsymbol{\beta}$ is multivariate normal.

$$\boldsymbol{\beta} \mid \ldots \sim N[(\mathbf{X}_1^\top\mathbf{X}_1)^{-1}\mathbf{X}_1^\top\mathbf{A}_1; (\mathbf{X}_1^\top\mathbf{X}_1)^{-1}\sigma_e^2]$$

where: $\mathbf{A}_1 = \mathbf{y} - \mathbf{Z}\mathbf{g} - \sum \lambda_k diag(\mathbf{Z}\,\alpha_k)\mathbf{X}_2\,\boldsymbol{\gamma}_k$.

b) Complete conditional a posteriori distribution for $\mathbf{g}$

$$P(\mathbf{g} \mid \ldots) \propto |\mathbf{I}\sigma_e^2|^{-\frac{1}{2}} exp\left\{-\frac{1}{2\sigma_e^2}(\mathbf{y} - \theta)^\top(\mathbf{y} - \theta)\right\} \exp\left\{-\frac{1}{2\sigma_g^2}\,\mathbf{g}^\top\mathbf{I}_g\,\mathbf{g}\right\}$$

$$P(\mathbf{g} \mid \ldots) \propto exp\left\{-\frac{1}{2\sigma_e^2}\left[(\mathbf{A}_2 - \mathbf{Z}\mathbf{g})^\top(\mathbf{A}_2 - \mathbf{Z}\mathbf{g}) + \frac{\sigma_e^2}{\sigma_g^2}(\mathbf{g}^\top\mathbf{I}_g\,\mathbf{g})\right]\right\}. \quad (2)$$

Considering and developing the quadratic form between the square brackets and disregarding the terms that do not depend on **g**, expression (2) can be rewritten as:

$$P(\mathbf{g}|\ldots) \propto exp\left\{-\frac{1}{2\sigma_e^2}\left[-2\ \mathbf{g}^\top\mathbf{Z}^\top\mathbf{A}_2 + \ \mathbf{g}^\top\left(\mathbf{Z}^\top\mathbf{Z} + \frac{\sigma_e^2}{\sigma_g^2}\mathbf{I}_g\right)\mathbf{g}\ \right]\right\}.$$

Through algebraic manipulation and completing squares concerning a multivariate normal distribution, we obtain:

$$P(\mathbf{g}|\ldots) \propto exp\left\{-\frac{1}{2\sigma_e^2}\left[\left[\mathbf{g} - \left(\mathbf{Z}^\top\mathbf{Z} + \frac{\sigma_e^2}{\sigma_g^2}\mathbf{I}_g\right)^{-1}\mathbf{Z}^\top\mathbf{A}_2\right]^\top\left(\mathbf{Z}^\top\mathbf{Z} + \frac{\sigma_e^2}{\sigma_g^2}\mathbf{I}_g\right)\times\right.\right.$$

$$\left.\left.\times\left[\mathbf{g} - \left(\mathbf{Z}^\top\mathbf{Z} + \frac{\sigma_e^2}{\sigma_g^2}\mathbf{I}_g\right)^{-1}\mathbf{Z}^\top\mathbf{A}_2\right]\right]\right\}.$$

Thus, the complete posterior distribution for the effect of genotype is multivariate Gaussian, given by:

$$\mathbf{g}|\ldots \sim N\left[\left(\mathbf{Z}^\top\mathbf{Z} + \frac{\sigma_e^2}{\sigma_\mathbf{g}^2}I_\mathbf{g}\right)^{-1}\mathbf{Z}^\top\mathbf{A}_2; \left(\mathbf{Z}^\top\mathbf{Z} + \frac{\sigma_e^2}{\sigma_\mathbf{g}^2}I_\mathbf{g}\right)^{-1}\sigma_e^2\right]$$

where: $\mathbf{A}_2 = \mathbf{y} - \mathbf{X}_{1\boldsymbol{\beta}} - \sum \lambda_k diag(Z\boldsymbol{\alpha}_k)\boldsymbol{X}_2\boldsymbol{\gamma}_k$.

c) Complete conditional a posteriori distribution for σ_g^2

$$P(\sigma_g^2|\ldots) \propto (\sigma_g^2)^{-\frac{n_g}{2}} exp\left\{-\frac{1}{2(\sigma_g^2)}\ \mathbf{g}^\top\mathbf{I}_g\ \mathbf{g}\right\}(\sigma_g^2)^{-1}$$

$$P(\sigma_g^2|\ldots) \propto (\sigma_g^2)^{-\left(\frac{n_g}{2}+1\right)} exp\left\{-\frac{n_g}{2(\sigma_g^2)n_g}\ \mathbf{g}^\top\mathbf{I}_g\ \mathbf{g}\right\}.$$

Thus, the complete a posteriori distribution for the variance of the genotype effect is an scaled inverted chi-squared, given by:

$$\sigma_g^2|\ldots \sim Esc - \chi^{-2}\left[n_g; \frac{\mathbf{g}^\top\mathbf{I}_g\ \mathbf{g}}{n_g}\right].$$

d) Complete conditional a posteriori distribution for λ_k

$$P(\lambda_k|\ldots) \propto exp\left\{-\frac{1}{2\sigma_e^2}(\mathbf{y} - \boldsymbol{\theta})^\top(\mathbf{y} - \boldsymbol{\theta})\right\}P(\lambda_k)$$

$$P(\lambda_k|\ldots) \propto exp\left\{-\frac{1}{2\sigma_e^2}(\mathbf{A}_4 - \lambda_k\phi_k)^\top(\mathbf{A}_4 - \lambda_k\phi_k) - \right.$$

$$\left. -\frac{1}{2\sigma_{\lambda_k}^2}(\lambda_k - \mu_{\lambda_k})^\top(\lambda_k - \mu_{\lambda_k})\right\} \quad (3)$$

where $\mu_{\lambda_k} = 0$.

Working with the terms between the keys of the expression (3), we have:

$$\mathbf{A}_4^\top\mathbf{A}_4 - \lambda_k\mathbf{A}_4^\top\phi_k - \lambda_k\phi_k^\top\mathbf{A}_4 - \lambda_k^2\phi_k^\top\phi_k + \frac{\sigma_e^2}{\sigma_{\lambda_k}^2}(\lambda_k^2 - \lambda_k^2\mu_{\lambda_k} + \mu_{\lambda_k}^2).$$

Furthermore, considering and developing the quadratic form and disregarding the terms that do not depend on λ_k, we have the following expression:

$$P(\lambda_k|\ldots) \propto exp\left\{-\frac{1}{2\sigma_e^2}\left(-2\lambda_k\phi_k^\top\mathbf{A}_4 + \lambda_k^2\left[\phi_k^\top\phi_k + \frac{\sigma_e^2}{\sigma_{\lambda_k}^2}\right]\right)\right\}.$$

Through algebraic manipulation and completing square concerning a normal distribution, we obtain:

$$\propto exp\left\{-\frac{1}{2\sigma_e^2}\left(\left[\lambda_k - \left(\phi_k^\top\phi_k + \frac{\sigma_e^2}{\sigma_{\lambda_k}^2}\right)^{-1}\phi_k\mathbf{A}_{4k}\right]^\top\left(\phi_k^\top\phi_k + \frac{\sigma_e^2}{\sigma_{\lambda_k}^2}\right)\right.\right.$$

$$\left.\left.\left[\lambda_k - \left(\phi_k^\top\phi_k + \frac{\sigma_e^2}{\sigma_{\lambda_k}^2}\right)^{-1}\phi_k\mathbf{A}_{4k}\right]\right)\right\}.$$

Thus, the complete posterior distribution for λ_k is given by a truncated normal (N^+):

$$\lambda_k|\ldots \sim N^+\left(\left[\left(\phi_k{}^\top\phi_k + \frac{\sigma_e^2}{\sigma_{\lambda_k}^2}\right)^{-1}\phi_k\mathbf{A}_{4k}\right]; \left(\phi_k{}^\top\phi_k + \frac{\sigma_e^2}{\sigma_{\lambda_k}^2}\right)^{-1}\sigma_e^2\right)$$

where: $\mathbf{A}_{4k} = \mathbf{y} - \mathbf{X}_1\boldsymbol{\beta} - \mathbf{Zg} - \sum_{k'\neq k}^{t-1}\lambda_{k'}diag(\mathbf{Z}\alpha_{k'})\mathbf{X}_2\boldsymbol{\gamma}_{k'}$ and $\phi_k = diag(\mathbf{Z}\boldsymbol{\alpha}_k)\mathbf{X}_2\gamma_k$.

e) Complete conditional a posteriori distribution for $\boldsymbol{\alpha}_k$

$$P(\boldsymbol{\alpha}_k|\ldots) \propto exp\left\{-\frac{1}{2\sigma_e^2}(\mathbf{y} - \theta)^\top(\mathbf{y} - \theta)\right\}P(\boldsymbol{\alpha}_k)$$

$$P(\boldsymbol{\alpha}_k|\ldots) \propto exp\left\{-\frac{1}{2\sigma_e^2}(\mathbf{A}_{4k} - \Delta_1\boldsymbol{\alpha}_k)^\top(\mathbf{A}_{4k} - \Delta_1\boldsymbol{\alpha}_k)\right\}. \quad (4)$$

Developing the calculation indicated in the numerator of expression (4) brackets, one obtains:

$$(\mathbf{A}_{4k} - \Delta_1 \boldsymbol{\alpha}_k)^\top (\mathbf{A}_{4k} - \Delta_1 \boldsymbol{\alpha}_k) = \mathbf{A}_{4k}^\top \mathbf{A}_{4k} - \mathbf{A}_{4k}^\top \Delta_1 \boldsymbol{\alpha}_k - \alpha_k^\top \Delta_1^\top \mathbf{A}_{4k} + \alpha_k^\top \Delta_1^\top \Delta_1 \boldsymbol{\alpha}_k.$$

Furthermore, considering and developing the quadratic form and disregarding the terms that do not depend on $\boldsymbol{\alpha}_k$, we have the following expression:

$$P(\boldsymbol{\alpha}_k|\ldots) \propto exp\left\{-\frac{1}{2\sigma_e^2}[-2\boldsymbol{\alpha}_k^\top \boldsymbol{\Delta}_1^\top (\mathbf{y} - \mathbf{X}_1\boldsymbol{\beta} - \mathbf{Zg})]\right\}$$

thus, it appears that $\boldsymbol{\alpha}_k$ has a distribution proportional to von Mises-Fisher (VMF) is represented as:

$$\boldsymbol{\alpha}_k|\ldots \sim VMF\left(\frac{\lambda_k}{\sigma_e^2};\ \Delta_1^\top (\mathbf{y} - \mathbf{X}_1\boldsymbol{\beta} - \mathbf{Zg})\right).$$

with directional mean $\Delta_{1k} = \lambda_k diag(\mathbf{X}_2 \boldsymbol{\gamma}_k)\mathbf{Z}$ and parameter concentration equal to λ_k/σ_e^2.

f) Complete conditional a posteriori distribution for $\boldsymbol{\gamma}_k$

$$P(\boldsymbol{\gamma}_k|\ldots) \propto exp\left\{-\frac{1}{2\sigma_e^2}(\mathbf{y} - \theta)^\top (\mathbf{y} - \theta)\right\} P(\boldsymbol{\gamma}_k)$$

$$P(\boldsymbol{\gamma}_k|\ldots) \propto exp\left\{-\frac{1}{2\sigma_e^2}(\mathbf{A}_4 - \Delta_2 \boldsymbol{\gamma}_k)^\top (\mathbf{A}_4 - \Delta_2 \boldsymbol{\gamma}_k)\right\}. \quad (5)$$

Analogously to that used for genotypic vectors, developing the calculations indicated in expression (5) between braces, we obtain:

$$(\mathbf{A}_4 - \Delta_2 \boldsymbol{\gamma}_k)^\top (\mathbf{A}_4 - \Delta_2 \boldsymbol{\gamma}_k) = \mathbf{A}_4^\top \mathbf{A}_4 - \mathbf{A}_4^\top \Delta_2 \boldsymbol{\gamma}_k - \boldsymbol{\gamma}_k^\top \Delta_2^\top \mathbf{A}_4 + \boldsymbol{\gamma}_k^\top \Delta_2^\top \Delta_2 \boldsymbol{\gamma}_k.$$

From algebraic manipulations in which terms not dependent on $\boldsymbol{\gamma}_k$ are absorbed by the normalization constant, the expression is rewritten as follows:

$$P(\boldsymbol{\gamma}_k|\ldots) \propto exp\left\{-\frac{1}{2\sigma_e^2}[-2\,\boldsymbol{\gamma}_k^\top \Delta_2^\top (\mathbf{y} - \mathbf{X}_1\boldsymbol{\beta} - \mathbf{Zg})]\right\}.$$

Thus, $\boldsymbol{\gamma}_k$ has a distribution proportional to a von Mises-Fisher (VMF) denoted by:

$$\boldsymbol{\gamma}_k|\ldots \sim VMF\left(\frac{\lambda_k}{\sigma_e^2};\ \Delta_2^{\top}(\mathbf{y}-\mathbf{X}_1\boldsymbol{\beta}\ -\mathbf{Zg}\,)\right)$$

with directional mean $\Delta_{2k} = \lambda_k diag(\mathbf{Z}\,\alpha_k)\mathbf{X}_2$ and parameter concentration equal to λ_k/σ_e^2.

g) Complete conditional a posteriori distribution for σ_e^2

$$P(\sigma_e^2|\ldots) \propto (\sigma_e^2)^{-\frac{n}{2}} exp\left\{-\frac{1}{2\sigma_e^2}(\mathbf{y}-\ \theta\,)^{\top}(\mathbf{y}-\ \theta\,)\right\}(\sigma_e^2)^{-1}.$$

Thus, the complete a posteriori distribution for the genotype error variance is an inverted scaled Chi-square, given by:

$$\sigma_e^2|\ldots \sim Esc-\chi^{-2}\left[n_e;\frac{(\mathbf{y}-\ \theta)^{\top}(\mathbf{y}-\ \theta)}{n_e}\right].$$

h) Complete conditional a posteriori distribution for ***t***

Given the prior distributions that correspond to parameter ***t***, the complete conditional posterior distribution for ***t*** is given by:

$$P(t|\ldots) \propto P(t|\mu)$$

$$P(t|\ldots) \propto \frac{\mu^t e^{-\mu}}{t!}$$

$$t \sim \text{truncated} - Poisson(\mu).$$

i) Complete conditional a posteriori distribution for μ:

Given the prior distributions that correspond to parameter μ, the complete conditional posterior distribution for μ is given by:

$$P(\mu|\ldots) \propto P(\mu)P(t|\mu)$$

$$P(\mu|\ldots) \propto \frac{\mu^t e^{-\mu}}{t!}\frac{\tau^{\nu}}{\Gamma(\nu)}\mu^{\nu-1}e^{-\tau\mu}$$

$$P(\mu|\ldots) \propto \mu^t e^{-2\mu}$$

$$\mu \sim gamma(\tau, \mu)$$

where: $\tau = 1$ and $v = 1$.

j) Complete conditional a posteriori distribution for $\sigma^2_{\lambda_k}$:

For the parameter $\sigma^2_{\lambda_k}$, as already mentioned, three hypotheses (prioris) were established, and they will have the corresponding complete a posteriori conditional distribution presented below.

Hypothesis i): The Bayes-Laplace insufficient reason principle is used, under the assumption that $\sigma^2_{\lambda_k} \to \infty$, what equivalent to assuming a constant prior distribution for λ_k. Under this hypothesis, the posterior distribution for λ_k reduces to:

$$\lambda_k | \ldots \sim N^+ \left(\left[\left({\phi_k}^\top \phi_k \right)^{-1} \phi_k \mathbf{A}_{4k} \right]; \left({\phi_k}^\top \phi_k \right)^{-1} \sigma^2_e \right).$$

Hypothesis ii): the assignment of a two-level hierarchical prior to λ_k with mean equal to zero and scaled inverser chi-squared distribution for $\sigma^2_{\lambda_k}$, with scale parameter equal to zero and degree of freedom $(1 - 2\Delta)$ as in Silva et al. [1]. The complete a posteriori conditional distribution for λ_k under this assumption is obtained from algebraic calculations as follows:

$$P(\sigma^2_{\lambda_k} | \ldots) \propto (\sigma^2_{\lambda_k})^{-\frac{1}{2}} exp \left\{ -\frac{1}{2(\sigma^2_{\lambda_k})} (\lambda_k - \mu_{\lambda_k})^\top (\lambda_k - \mu_{\lambda_k}) \right\} P(\sigma^2_{\lambda_k})$$

$$P(\sigma^2_{\lambda_k} | \ldots) \propto (\sigma^2_{\lambda_k})^{-\frac{1}{2}} exp \left\{ -\frac{1}{2(\sigma^2_{\lambda_k})} (\lambda_k - \mu_{\lambda_k})^\top (\lambda_k - \mu_{\lambda_k}) \right\} (\sigma^2_{\lambda_k})^{(\Delta - 1)}$$

$$P(\sigma^2_{\lambda_k} | \ldots) \propto (\sigma^2_{\lambda_k})^{\frac{1}{2} + (\Delta - 1)} exp \left\{ -\frac{1}{2(\sigma^2_{\lambda_k})} \lambda^2_k \right\}$$

$$P(\sigma^2_{\lambda_k} | \ldots) \propto (\sigma^2_{\lambda_k})^{-\frac{(1-2\Delta)}{2} - 1} exp \left\{ -\frac{(1 - 2\Delta)}{2(\sigma^2_{\lambda_k})} \frac{\lambda^2_k}{(1 - 2\Delta)} \right\}$$

$$\sigma^2_{\lambda_k} | \ldots \sim Esc - \chi^{-2} \left[(1 - 2\Delta), \frac{\lambda^2_k}{(1 - 2\Delta)} \right].$$

Hypothesis iii) Using the principle of maximum entropy in the prior specification obtaining an inverse gamma range for $\sigma^2_{\lambda_k}$, that is $\sigma^2_{\lambda_k} \sim Inv - gamma(a, b)$, wherein

$a = 1$ and $b = 0$. Under this assumption, the complete a posteriori conditional distribution for $\sigma^2_{\lambda_k}$, like the other parameters, is obtained by algebraic manipulations from the joint posterior distribution and given by:

$$P(\sigma^2_{\lambda_k}| \ldots) \propto (\sigma^2_{\lambda_k})^{-\frac{1}{2}} exp\left\{-\frac{1}{2\left(\sigma^2_{\lambda_k}\right)}(\lambda_k - \mu_{\lambda_k})^{\top}(\lambda_k - \mu_{\lambda_k})\right\} P(\sigma^2_{\lambda_k})$$

$$P(\sigma^2_{\lambda_k}|\ldots) \propto (\sigma^2_{\lambda_k})^{-\frac{1}{2}} exp\left\{-\frac{1}{2(\sigma^2_{\lambda_k})}(\lambda_k - \mu_{\lambda_k})^{\top}(\lambda_k - \mu_{\lambda_k})\right\} \times$$

$$\times (\sigma^2_{\lambda_k})^{-\left(v_{\lambda_k}-1\right)} exp\left\{-\frac{S^2_{\lambda_k}}{\sigma^2_{\lambda_k}}\right\}$$

$$P(\sigma^2_{\lambda_k}|\ldots) \propto (\sigma^2_{\lambda_k})^{-\left[\frac{\left(2v_{\lambda_k}+1\right)}{2}-1\right]} exp\left\{-\frac{S^2_{\lambda_k} + \lambda^2_k}{2(\sigma^2_{\lambda_k})}\right\}$$

$$\sigma^2_{\lambda_k}|\ldots \sim inv - gama\left(\frac{2v_{\lambda_k} + 1}{2}; \frac{\left[S_{\lambda_k} + \lambda^2_k\right]}{2}\right).$$

References

1. da Silva CP, de Oliveira LA, Nuvunga JJ, Pamplona AKA, Balestre M. A Bayesian Shrinkage Approach for AMMI Models. Hsiao CK, editor. PLoS One [Internet]. 2015;10(7):e0131414. Available from: https://dx.plos.org/10.1371/journal.pone.0131414
