# Supplementary material for: Use of the reversible jump Markov chain Monte Carlo algorithm to select multiplicative terms in the AMMI-Bayesian model
Source: PLoS One. 2023 Jan 3;18(1):e0279537. doi: 10.1371/journal.pone.0279537 (PMC9810207; doi:10.1371/journal.pone.0279537)
Supplement: S4 Appendix — (PDF) [file pone.0279537.s004.pdf]

S4 Appendix

Posterior summaries for AMMI models with the MCMC method selected by AIC and BIC

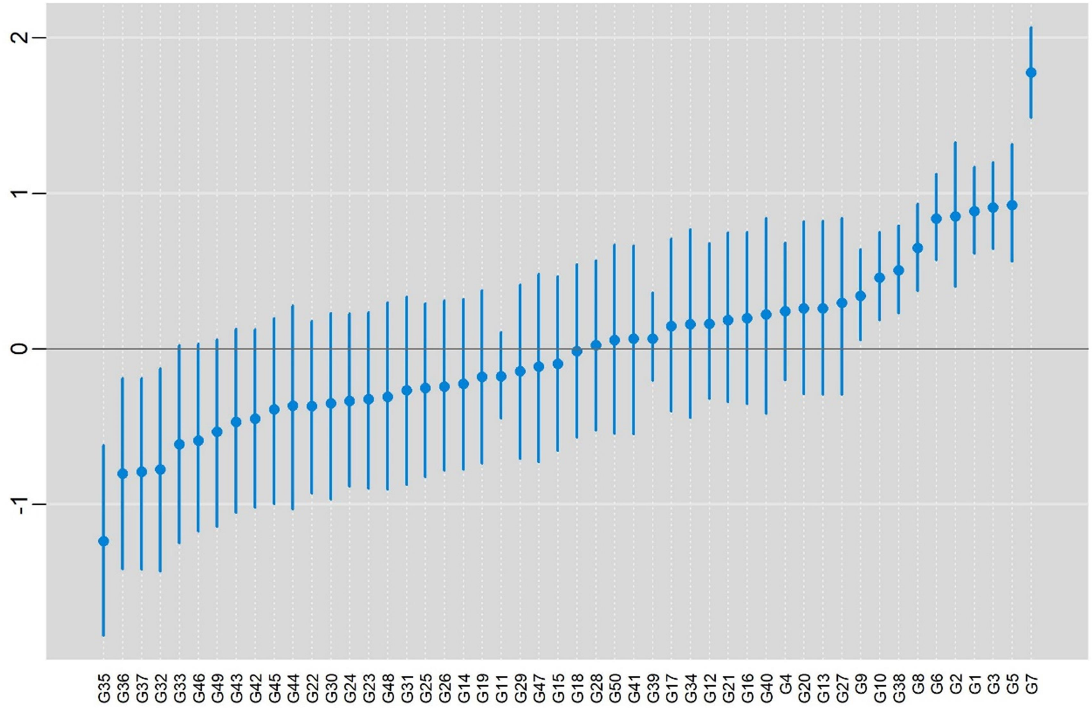

S7 Fig. Posterior means and the 95% HPD intervals for the effect of genotypes, considering the BAMMIS-4 model, for real data using the MCMC method.

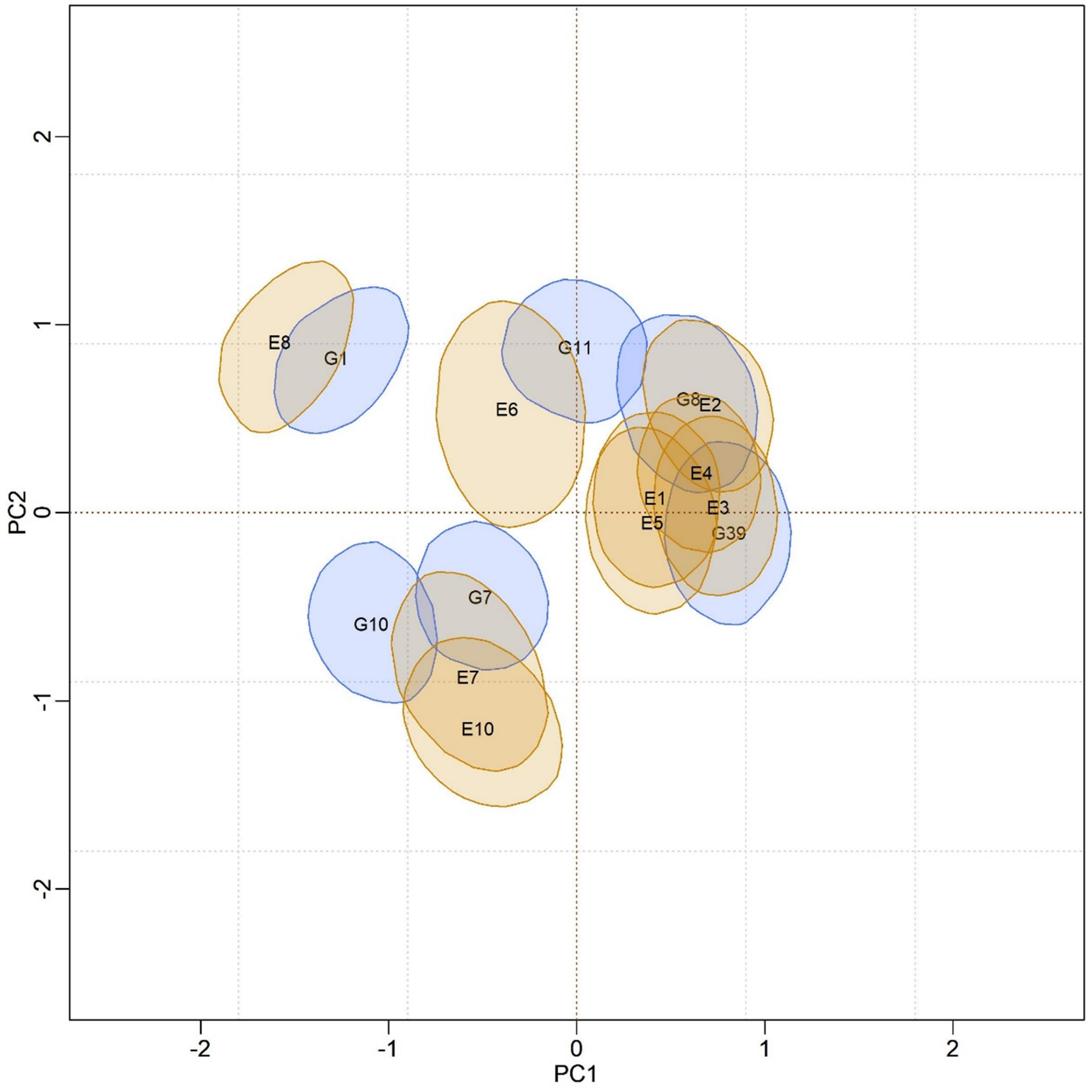

S8 Fig. Bivariate credibility regions at 95% probability for the genotypic and environmental scores of the BAMMIS-4 model for real data, adjusted using the MCMC method. Only regions whose regions do not contain the origin (0.0) were plotted.

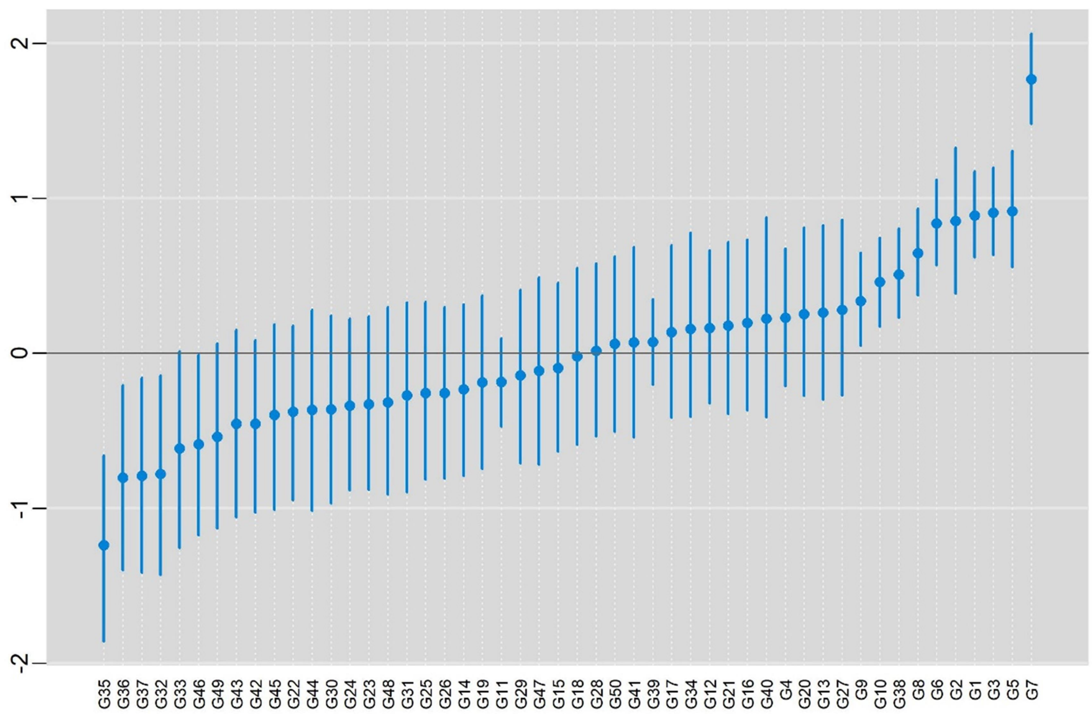

S9 Fig. Posterior means and the 95% HPD intervals for the effect of genotypes, considering the BAMMIS-5 model, for real data using the MCMC method.

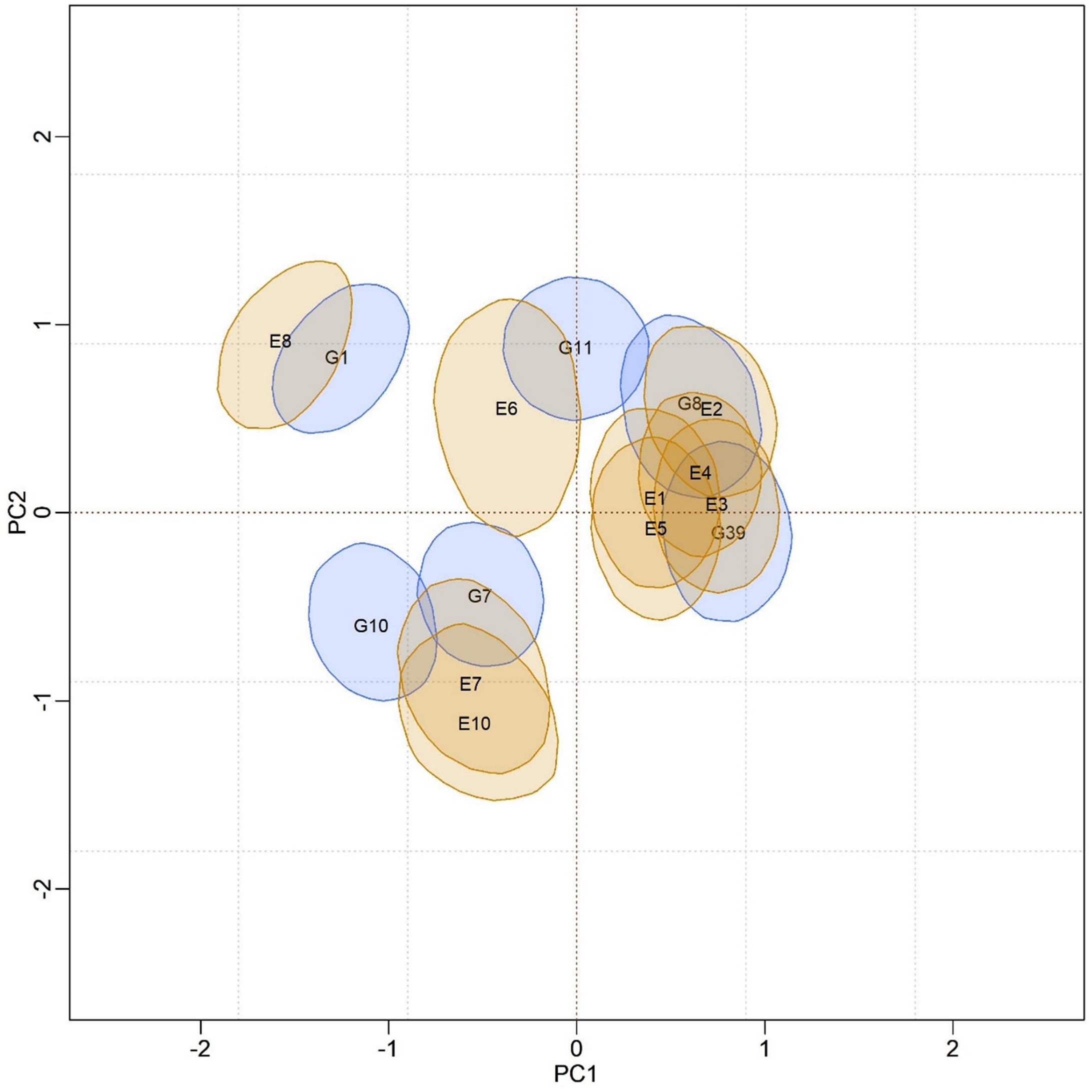

S10 Fig. Bivariate credibility regions at 95% probability for the genotypic and environmental scores of the BAMMIS-5 model for real data, adjusted using the MCMC method. Only regions whose regions do not contain the origin (0.0) were plotted.

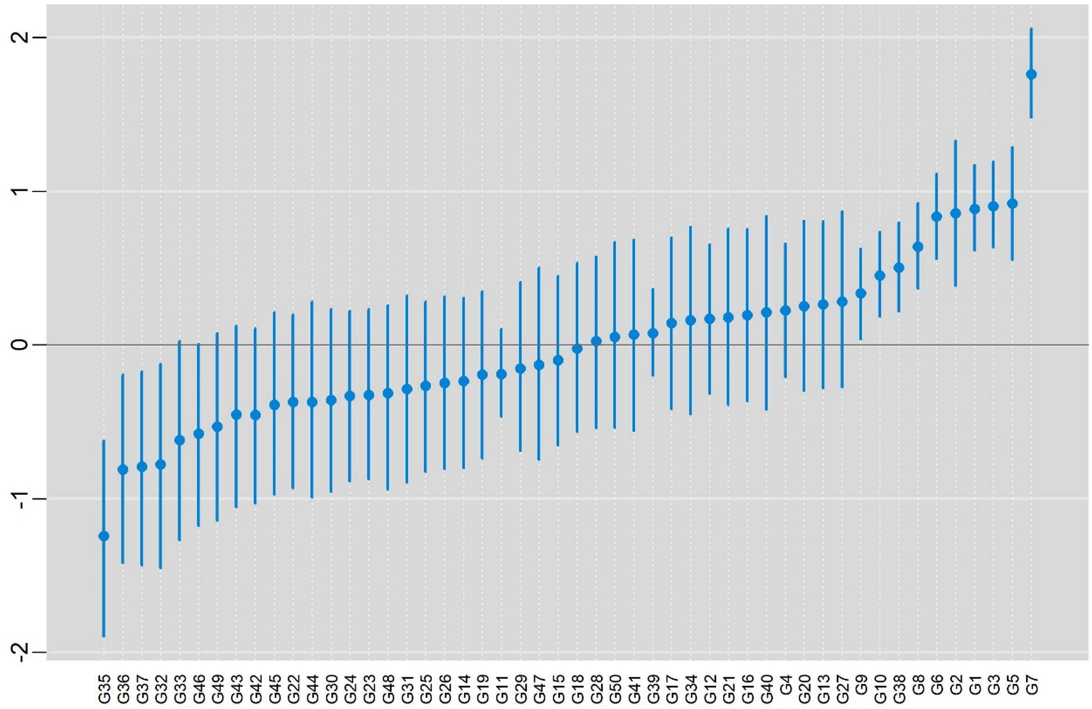

S11 Fig. Posterior means and the 95% HPD intervals for the effect of genotypes, considering the BAMMIS-5 model, for real data using the MCMC method.

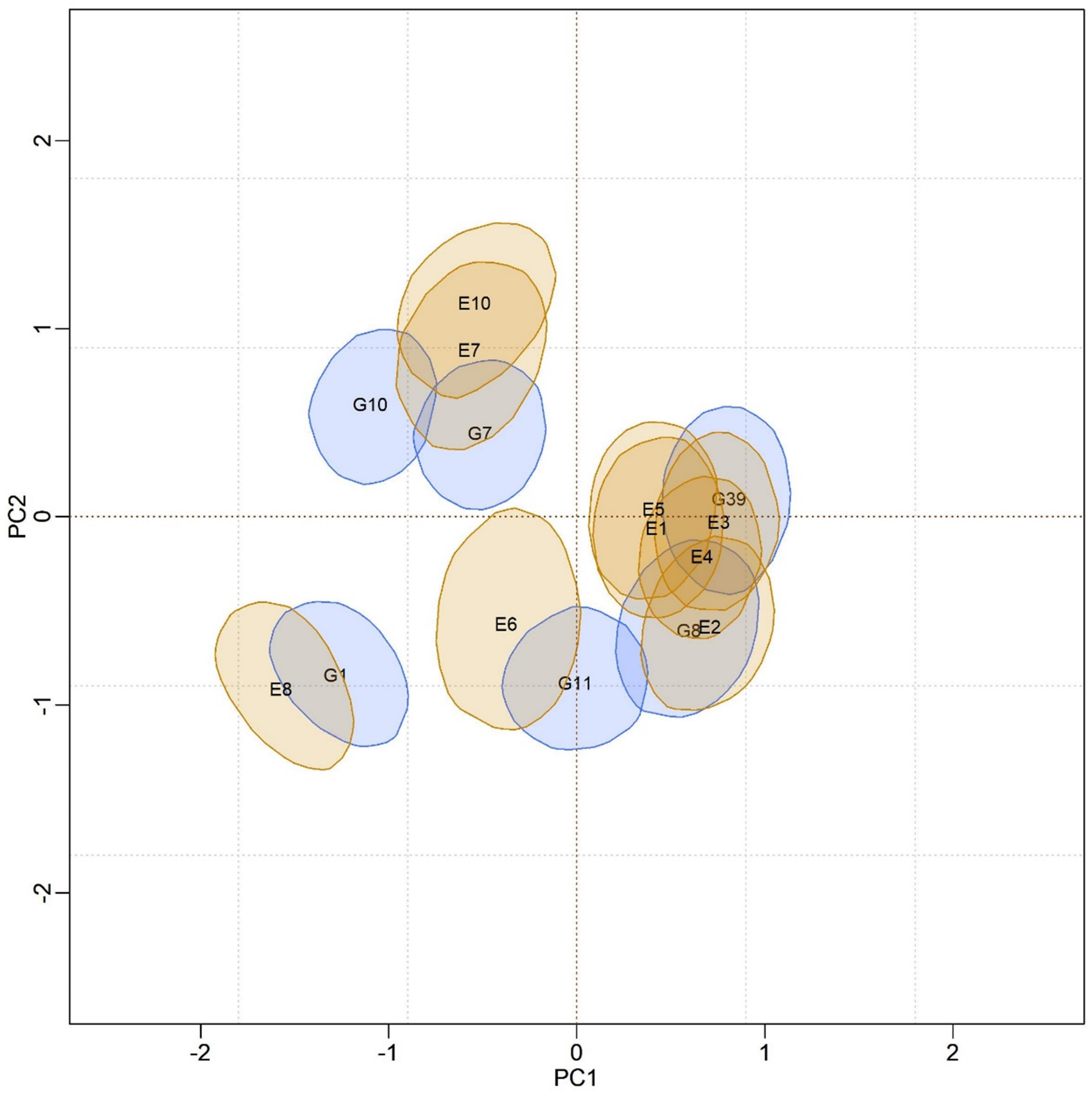

S12 Fig. Bivariate credibility regions at 95% probability for the genotypic and environmental scores of the BAMMI-5 model for real data, adjusted using the MCMC method. Only regions whose regions do not contain the origin (0.0) were plotted.
