# Supplementary material for: Use of the reversible jump Markov chain Monte Carlo algorithm to select multiplicative terms in the AMMI-Bayesian model
Source: PLoS One. 2023 Jan 3;18(1):e0279537. doi: 10.1371/journal.pone.0279537 (PMC9810207; doi:10.1371/journal.pone.0279537)
Supplement: S1 Table — (PDF) [file pone.0279537.s005.pdf]

S1 Table. Singular values were obtained through the decomposition of singular values of the simulated interaction matrix, and together with the percentage contribution of each main axis to the GEI and also the accumulated in each.

Dim.	λ_k	λ^2	λ^2(%)	Cum	Cum. (%)
1	11.82	139.769	0.538	139.769	0.54
2	6.56	43.074	0.166	182.843	0.70
3	5.03	25.348	0.098	208.192	0.80
4	4.17	17.354	0.067	225.546	0.87
5	3.70	13.660	0.053	239.206	0.92
6	3.25	10.556	0.041	249.762	0.96
7	2.63	6.893	0.027	256.655	0.99
8	1.72	2.949	0.011	259.604	1.00

Dim = dimension, Cum = cumulated.
