# Supplementary material for: Use of the reversible jump Markov chain Monte Carlo algorithm to select multiplicative terms in the AMMI-Bayesian model
Source: PLoS One. 2023 Jan 3;18(1):e0279537. doi: 10.1371/journal.pone.0279537 (PMC9810207; doi:10.1371/journal.pone.0279537)
Supplement: S2 Table — (PDF) [file pone.0279537.s006.pdf]

S2 Table. Point estimates for the variance components and the genotypic effect variance, referring to the three approaches given to the AMMI model. Using Gibbs algorithm.

Mod	Dim.	σ_e^2				σ_g^2			
		Mean	Sd	LL	UL	Mean	Sd	LL	UL
BAMMI	1	3.433	0.223	5.055	20.530	11.077	4.437	5.055	20.529
	2	**2.958**%	0.196	2.589	3.357	11.148	4.444	5.320	20.733
	3	**2.753**&	0.197	2.403	3.149	11.077	4.337	5.638	20.724
	4	**2.689***	0.201	2.318	3.105	11.042	4.308	5.702	20.956
	5	2.711	0.210	2.342	3.159	11.000	4.428	5.053	20.383
	6	2.722	0.209	2.346	3.149	11.179	4.487	5.606	21.289
	7	2.737	0.211	2.337	3.152	10.977	4.369	5.218	20.451
	8	2.733	0.209	2.338	3.155	11.084	4.507	5.269	20.820
BAMMIS	1	3.435	0.225	2.983	3.871	11.146	4.333	5.499	20.783
	2	**2.96**%	0.204	2.587	3.369	11.238	4.395	5.266	20.737
	3	**2.76***&	0.195	2.411	3.171	11.154	4.315	5.19	20.54
	4	2.775	0.238	2.346	3.252	11.176	4.550	5.206	20.675
	5	2.865	0.221	2.442	3.302	11.122	4.255	5.539	20.587
	6	2.864	0.221	2.441	3.293	11.088	4.289	5.14	20.16
	7	2.869	0.226	2.458	3.328	11.091	4.406	5.383	20.439
	8	2.870	0.224	2.459	3.325	11.104	4.398	5.242	20.603
BAMMIE	1	3.438	0.227	3.001	3.896	11.079	4.527	5.337	20.557
	2	**2.96***%&	0.201	2.581	3.366	11.118	4.539	5.317	21.446
	3	3.428	0.238	2.963	3.913	10.962	4.321	5.257	20.244
	4	3.428	0.239	2.945	3.899	11.038	4.455	5.129	20.495
	5	3.433	0.239	2.974	3.922	11.125	4.482	5.296	20.577
	6	3.418	0.249	2.932	3.933	11.045	4.536	5.265	21.006
	7	3.427	0.242	2.976	3.924	11.075	4.465	5.326	20.717
	8	3.447	0.228	3.003	3.897	12.003	4.476	5.26	20.452

The winning models based on information criteria are designated by: "*" AIC; “&” BIC and “%” AICM, Sd = upper limit, LL = lower limit and UL = upper limit.
