# Supplementary material for: Use of the reversible jump Markov chain Monte Carlo algorithm to select multiplicative terms in the AMMI-Bayesian model
Source: PLoS One. 2023 Jan 3;18(1):e0279537. doi: 10.1371/journal.pone.0279537 (PMC9810207; doi:10.1371/journal.pone.0279537)
Supplement: S3 Table — (PDF) [file pone.0279537.s007.pdf]

S3 Table. Posterior means and HPD intervals (at 95% of credibility) for the genotypic effects, for the conditional and marginal responses of the BAMMI model (AMMI3).

	Conditional			Marginal		
Gen.	Mean	LL	UL	Mean	LL	UL
G1	2.3076	0.6214	3.9259	2.3041	0.6162	3.9388
G2	-4.2906	-6.0000	-2.6143	-4.2994	-5.9613	-2.6143
G3	-0.7547	-2.3944	0.9580	-0.7551	-2.4082	0.9487
G4	3.8307	2.0673	5.4454	3.8230	2.1555	5.5205
G5	-2.6039	-4.3413	-0.9965	-2.6074	-4.3154	-0.9471
G6	-1.1793	-2.9050	0.4380	-1.1813	-2.8959	0.4271
G7	0.6503	-0.9661	2.3024	0.6504	-0.9592	2.3860
G8	0.5387	-1.1076	2.3032	0.5334	-1.0954	2.2990
G9	-3.5352	-5.2939	-1.9268	-3.5394	-5.3327	-1.9399
G10	3.9510	2.3028	5.6735	3.9479	2.3028	5.6836
G11	5.9657	4.3418	7.6942	5.9612	4.1554	7.5331
G12	3.7171	2.0483	5.3837	3.7208	2.0365	5.4009
G13	4.3909	2.7733	6.0759	4.3869	2.7277	6.0655
G14	-2.0399	-3.6915	-0.3521	-2.0432	-3.6216	-0.2895
G15	1.4976	-0.1610	3.1490	1.4970	-0.1531	3.1748
G16	-3.6424	-5.2962	-1.9753	-3.6448	-5.3188	-1.9753
G17	0.3446	-1.3072	2.0240	0.3447	-1.3111	2.0420
G18	-2.8979	-4.6051	-1.2394	-2.9004	-4.6086	-1.2490
G19	-5.2065	-6.8231	-3.4170	-5.2125	-6.9465	-3.5473
G20	-1.2811	-3.0541	0.3816	-1.2832	-2.9417	0.4753

LL = lower limit and UL = upper limit.
