# Supplementary material for: Use of the reversible jump Markov chain Monte Carlo algorithm to select multiplicative terms in the AMMI-Bayesian model
Source: PLoS One. 2023 Jan 3;18(1):e0279537. doi: 10.1371/journal.pone.0279537 (PMC9810207; doi:10.1371/journal.pone.0279537)
Supplement: S4 Table — (PDF) [file pone.0279537.s008.pdf]

S4 Table. Posterior means and HPD intervals (at 95% of credibility) for the genotypic effects, for the conditional and marginal responses of the BAMMIS model (AMMI3).

	Conditional			Marginal		
Gen.	Mean	LL	UL	Mean	LL	UL
G1	2.3114	0.6842	3.9948	2.3143	0.7138	4.0356
G2	-4.2969	-5.8495	-2.6065	-4.2863	-5.8741	-2.6254
G3	-0.7452	-2.3312	0.9947	-0.7382	-2.3473	0.9756
G4	3.8311	2.1339	5.4486	3.8406	2.2291	5.5549
G5	-2.6103	-4.2141	-0.9774	-2.6027	-4.2206	-0.9774
G6	-1.1753	-2.7127	0.6196	-1.1643	-2.7602	0.5376
G7	0.6568	-0.9981	2.3626	0.6700	-1.0290	2.3249
G8	0.5414	-1.1303	2.2089	0.5560	-1.1279	2.2095
G9	-3.5288	-5.1106	-1.8403	-3.5228	-5.2240	-1.9176
G10	3.9629	2.2467	5.5759	3.9663	2.3013	5.6334
G11	5.9743	4.3784	7.7055	5.9833	4.3462	7.6585
G12	3.7314	2.0599	5.3234	3.7370	2.0599	5.3331
G13	4.4067	2.8009	6.0859	4.4056	2.7956	6.0949
G14	-2.0387	-3.7136	-0.4307	-2.0261	-3.6334	-0.3477
G15	1.5044	-0.0964	3.2216	1.5180	-0.0995	3.1830
G16	-3.6410	-5.2279	-1.9206	-3.6339	-5.3038	-1.9681
G17	0.3517	-1.3504	1.9632	0.3559	-1.3740	1.9588
G18	-2.8874	-4.5102	-1.2513	-2.8822	-4.5265	-1.2571
G19	-5.2048	-6.8430	-3.4962	-5.1996	-6.7997	-3.4706
G20	-1.2709	-2.8956	0.3985	-1.2633	-2.8877	0.4205

LL = lower limit and UL = upper limit.
