# Supplementary material for: Use of the reversible jump Markov chain Monte Carlo algorithm to select multiplicative terms in the AMMI-Bayesian model
Source: PLoS One. 2023 Jan 3;18(1):e0279537. doi: 10.1371/journal.pone.0279537 (PMC9810207; doi:10.1371/journal.pone.0279537)
Supplement: S5 Table — (PDF) [file pone.0279537.s009.pdf]

S5 Table. Posterior means and HPD intervals (at 95% of credibility) for the genotypic effects, for the conditional and marginal responses of the BAMMIE model (AMMI2).

	Conditional			Marginal		
Gen.	Mean	LL	UL	Mean	LL	UL
G1	2.2943	0.5728	3.9539	2.2958	0.5771	3.9343
G2	-4.3083	-6.0147	-2.5553	-4.3048	-6.0290	-2.5980
G3	-0.7602	-2.4000	1.0279	-0.7637	-2.4741	0.9234
G4	3.8139	2.0032	5.4546	3.8127	2.1224	5.5062
G5	-2.6277	-4.2318	-0.8227	-2.6258	-4.3294	-0.9568
G6	-1.1901	-2.8991	0.5450	-1.1844	-2.8898	0.5199
G7	0.6466	-1.0278	2.4284	0.6466	-1.1320	2.2881
G8	0.5237	-1.2759	2.2116	0.5240	-1.2600	2.2124
G9	-3.5504	-5.2802	-1.8556	-3.5464	-5.3332	-1.9530
G10	3.9432	2.2326	5.6365	3.9410	2.2326	5.6067
G11	5.9487	4.1586	7.6092	5.9525	4.1979	7.5909
G12	3.7083	1.9984	5.4686	3.7062	1.9833	5.3911
G13	4.3856	2.7999	6.2159	4.3845	2.6294	6.0117
G14	-2.0419	-3.7523	-0.2517	-2.0422	-3.7274	-0.2724
G15	1.4886	-0.2357	3.2118	1.4915	-0.2737	3.1121
G16	-3.6523	-5.3489	-1.8894	-3.6533	-5.3489	-1.9472
G17	0.3288	-1.3952	2.0459	0.3339	-1.3952	2.0186
G18	-2.9104	-4.6059	-1.2256	-2.9125	-4.6125	-1.2469
G19	-5.2230	-7.1012	-3.6650	-5.2212	-6.8847	-3.4943
G20	-1.2826	-2.9213	0.5048	-1.2842	-2.8635	0.5048

LL = lower limit and UL = upper limit.
