# Supplementary material for: Use of the reversible jump Markov chain Monte Carlo algorithm to select multiplicative terms in the AMMI-Bayesian model
Source: PLoS One. 2023 Jan 3;18(1):e0279537. doi: 10.1371/journal.pone.0279537 (PMC9810207; doi:10.1371/journal.pone.0279537)
Supplement: S6 Table — (PDF) [file pone.0279537.s010.pdf]

S6 Table. Correlation and PRESS information for evaluating the predictive capacity of the models (Reversible Jump).

	BAMMI		BAMMIS		BAMMIE	
Desb.	Cor	Press	Cor	Press	Cor	Press
1	0.9062	2.5688	0.9053	2.5941	0.8966	2.8199
2	0.9052	1.8575	0.9041	1.8785	0.8854	2.2149
3	0.8964	2.4518	0.8937	2.5061	0.8404	3.6766
4	0.9499	1.6744	0.9490	1.7055	0.9342	2.3175
5	0.9173	2.6461	0.9160	2.6913	0.9010	3.3667
6	0.9300	2.7205	0.9295	2.7585	0.9088	3.4480
7	0.9081	2.8939	0.9071	2.9307	0.8810	3.6857
8	0.9544	1.6925	0.9541	1.7080	0.9462	1.9956
9	0.9212	2.1946	0.9199	2.2376	0.8948	3.0070
10	0.9378	1.6982	0.9364	1.7305	0.9094	2.4309
Mean	0.9227	2.2398	0.9215	2.2741	0.8998	2.8963
