# Supplementary material for: Use of the reversible jump Markov chain Monte Carlo algorithm to select multiplicative terms in the AMMI-Bayesian model
Source: PLoS One. 2023 Jan 3;18(1):e0279537. doi: 10.1371/journal.pone.0279537 (PMC9810207; doi:10.1371/journal.pone.0279537)
Supplement: S7 Table — (PDF) [file pone.0279537.s011.pdf]

S7 Table. Total computational time, in minutes, for the sampling process of the three Bayesian-AMMI versions considering adjustment by information criteria and by the RJMCMC method.

Model	Time	
	Gibbs/Critérios de informação	RJMCMC
BAMMI	887.6 mins	210.19 mins
BAMMIE	913.87 mins	190.24 mins
BAMMIS	905.68 mins	198.84 mins
