# Supplementary material for: Use of the reversible jump Markov chain Monte Carlo algorithm to select multiplicative terms in the AMMI-Bayesian model
Source: PLoS One. 2023 Jan 3;18(1):e0279537. doi: 10.1371/journal.pone.0279537 (PMC9810207; doi:10.1371/journal.pone.0279537)
Supplement: S9 Table — (PDF) [file pone.0279537.s013.pdf]

S9 Table. Classification of the pattern of genotypic scores by biplot AMMI2 representation based on the simulated scenario (RJMCMC algorithm).

Unstable										
Model	G1	G2	G3	G4	G5	G6	G7	G8	G9	G10
BAMMI	T	F	T	F	T	T	T	T	T	T
BAMMIE	T	F	F	F	T	T	F	T	T	T
BAMMIS	T	F	F	F	T	T	T	T	T	T
Stable										
Model	G11	G12	G13	G14	G15	G16	G17	G18	G19	G20
BAMMI	F	F	F	F	T	F	T	T	T	T
BAMMIE	F	F	T	F	T	T	T	T	T	T
BAMMIS	F	F	T	F	T	T	T	T	T	T

T= True and F=False.
