# Supplementary material for: Use of the reversible jump Markov chain Monte Carlo algorithm to select multiplicative terms in the AMMI-Bayesian model
Source: PLoS One. 2023 Jan 3;18(1):e0279537. doi: 10.1371/journal.pone.0279537 (PMC9810207; doi:10.1371/journal.pone.0279537)
Supplement: S1 Fig — (PDF) [file pone.0279537.s014.pdf]

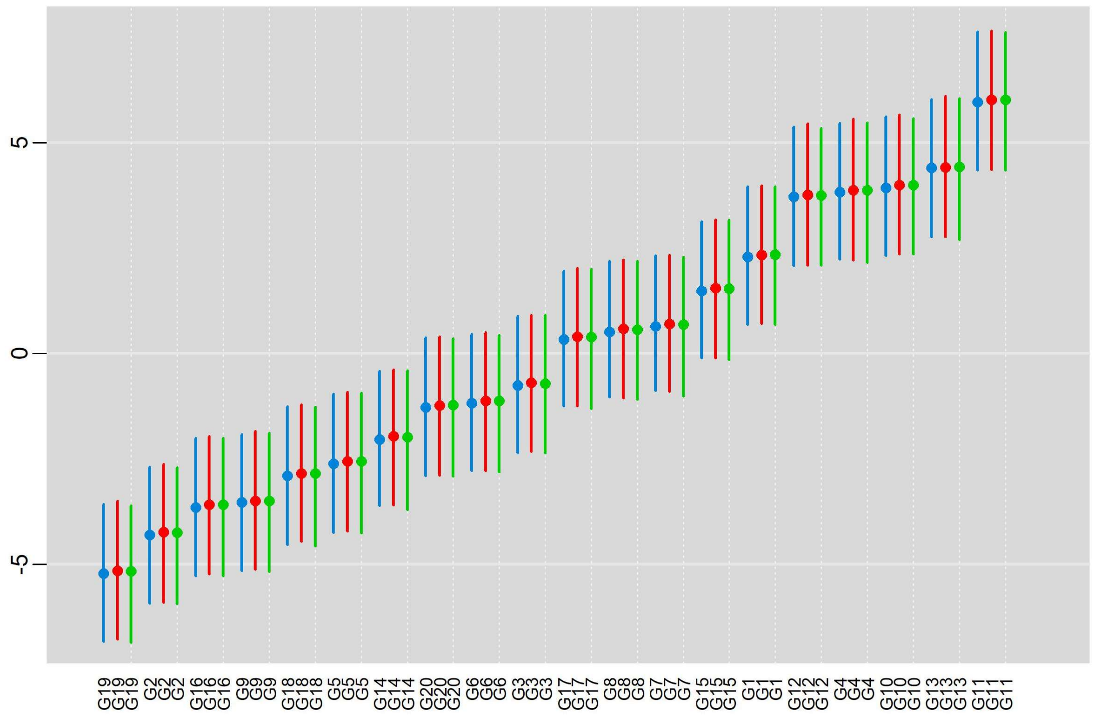

S1 Fig. Genotypic effect of the BAMMI model based on the adjustment by the information criteria BIC (AMMI-3), AIC (AMMI-4), and AICM (AMMI2) (Gibbs algorithm).
