# Supplementary material for: Use of the reversible jump Markov chain Monte Carlo algorithm to select multiplicative terms in the AMMI-Bayesian model
Source: PLoS One. 2023 Jan 3;18(1):e0279537. doi: 10.1371/journal.pone.0279537 (PMC9810207; doi:10.1371/journal.pone.0279537)
Supplement: S3 Fig — (PDF) [file pone.0279537.s016.pdf]

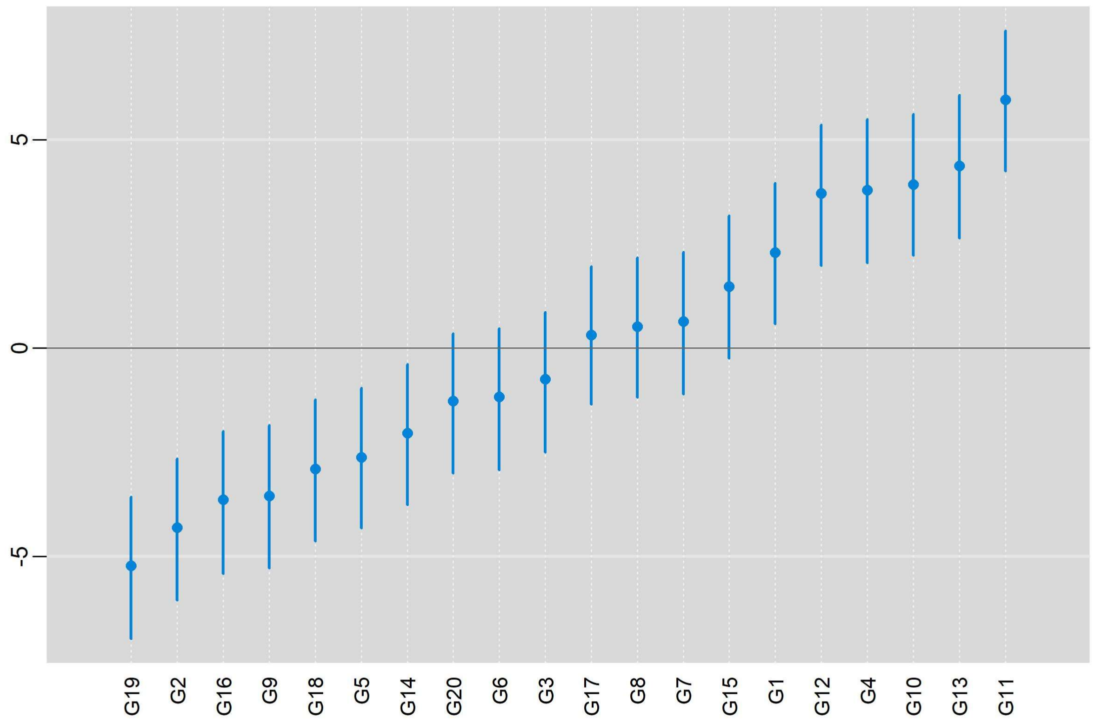

S3 Fig. Genotypic effect of the BAMMIE model based on adjustment by BIC/AIC/AICM (AMMI2) information criteria (Gibbs algorithm).
