# Supplementary material for: Use of the reversible jump Markov chain Monte Carlo algorithm to select multiplicative terms in the AMMI-Bayesian model
Source: PLoS One. 2023 Jan 3;18(1):e0279537. doi: 10.1371/journal.pone.0279537 (PMC9810207; doi:10.1371/journal.pone.0279537)
Supplement: S5 Fig — (PDF) [file pone.0279537.s018.pdf]

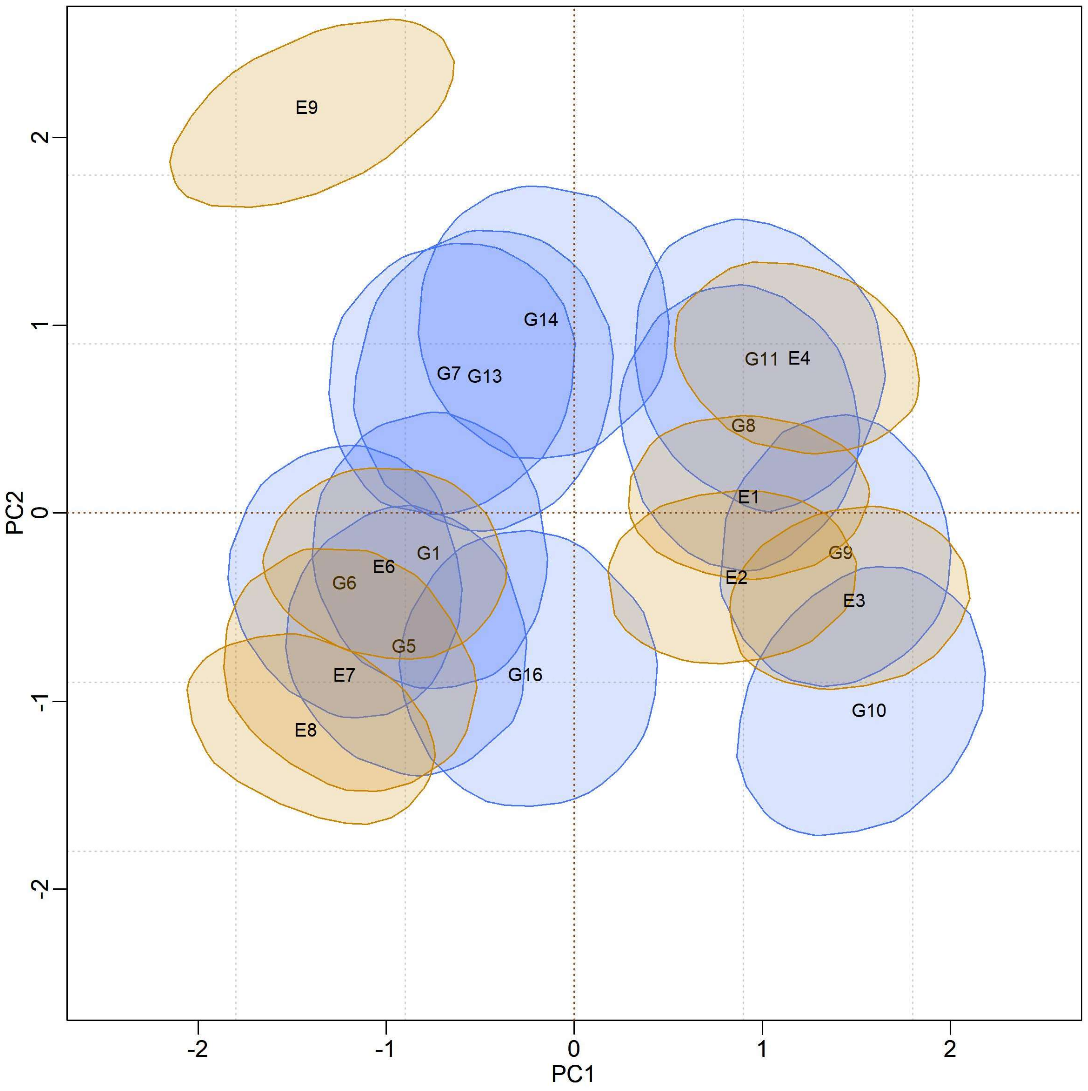

S5 Fig. Bivariate credibility regions for the genotypic and environmental scores of the BAMMIE-2 model, whose regions do not include the origin (0.0) (Gibbs algorithm).
